# New species of *Protomicrocotyle* (Monogenea: Protomicrocotylidae), and new information on *P. mirabilis*, parasites of *Caranx* spp. from Veracruz, México

**DOI:** 10.1590/S1984-29612023066

**Published:** 2023-11-27

**Authors:** Edgar Salvador Ramírez-Cruz, Scott Monks, Norma Leticia Manríquez-Morán, Juan Violante-González, Griselda Pulido-Flores

**Affiliations:** 1 Laboratorio de Morfología Animal, Centro de Investigaciones Biológicas, Instituto de Ciencias Básicas e Ingeniería, Universidad Autónoma del Estado de Hidalgo, Pachuca, Hidalgo, México; 2 Laboratorio de Sistemática Molecular, Centro de Investigaciones Biológicas, Instituto de Ciencias Básicas e Ingeniería, Universidad Autónoma del Estado de Hidalgo, Pachuca, Hidalgo, México; 3 Centro de Ciencias de Desarrollo Regional, Facultad de Ecología Marina, Universidad Autónoma de Guerrero, Acapulco, Guerrero, Mexico

**Keywords:** Monogenea, marine fishes, *Protomicrocotyle veracruzensis* sp. nov., CO1, 28S, neighbor-joining, Monogenea, peixes marinhos, *Protomicrocotyle veracruzensis* sp. nov., CO1, 28S, associação de vizinho

## Abstract

During a study of the helminth parasites of carangid fish of the Gulf of Mexico, *Protomicrocotyle mirabilis* and a new member of that genus were found. The aim of the present study is to provide new morphological and sequences of 28s rDNA and CO1 mtDNA for *P. mirabilis* and describe the new species. Between 2005–2022, 73 specimens of *Caranx* spp. were purchased from local fishermen of the littoral waters of the Gulf of Mexico. *Protomicrocotyle veracruzensis* sp. nov. is most similar to *P*. *mirabilis* than to *P*. *ivoriensis*, the only members of the genus known from the Greater Atlantic Ocean Basin. *Protomicrocotyle veracruzensis* sp. nov. can be distinguished from those two species by the arrangement and number of testes. Measurement data on the haptoral armature for the new species is provided and the potential value and need for comparative data from these structures of other members of the genus is discussed. The results of the molecular analysis and the morphometric analysis of 91 characters confirmed that this new species belongs to *Protomicrocotyle*.

## Resumo

Encontraram-se durante um estudo dos helmintos parasitas de peixes carangídeos do Golfo do México, *Protomicrocotyle mirabilis* e um novo membro do gênero. O objetivo do presente estudo é fornecer novos dados morfológicos e sequências de 28s rDNA e CO1 mtDNA para *P. mirabilis* e descrever a nova espécie. Entre 2005-2022, 73 espécimes de *Caranx* spp. foram comprados de pescadores locais das águas litorâneas do Golfo do México. *Protomicrocotyle veracruzensis* sp. nov. é mais semelhante a *P. mirabilis* do que a *P. ivoriensis*, os únicos membros do género conhecidos da Grande Bacia do Oceano Atlântico. *Protomicrocotyle veracruzensis* sp. nov. pode ser distinguido dessas duas espécies pela disposição e número de testículos. Neste estudo são fornecidos dados de medidas da armadura haptoral para a nova espécie, discutido o valor potencial e a necessidade de dados comparativos destas estruturas de outros membros do gênero. Os resultados da análise molecular e da análise morfométrica de 91 caracteres confirmaram que essa nova espécie pertence ao gênero *Protomicrocotyle*.

## Introduction

The Carangidae is one of the most morphologically diverse families of fishes (Nelson, 2006). The family is comprised of approximately 140 species, that are assigned to four tribes of 32 genera (Jacobina et al., 2014). Systematic analyses of the genus *Caranx* Lacépède, 1801 has shown that some recognized species have wide geographic distribution and regional populations with cryptic taxonomic features indicating that they constitute species complexes (Jacobina et al., 2014). In the Gulf of Mexico, the most common species of *Caranx* are *Caranx hippos*, *Caranx crysos*, and *Caranx latus* (López-Herrera et al., 2021). However, there are some registers of *Caranx ruber*, *Caranx bartholomaei,* and *Caranx lugubris* (Froese & Pauly, 2022).

Members of the family Carangidae are usual hosts for monogenean parasites. Several species of Protomicrocotylidae Johnston & Tiegs, 1922 commonly infect the gills of carangid fishes (Poche, 1926; Yamaguti, 1963; Mendoza-Garfias et al., 2017). *Protomicrocotyle* was establish by Johnston & Tiegs (1922), who transferred *Acanthodiscus mirabile* MacCallum, 1918 as *Protomicrocotyle mirabilis* (MacCallum, 1918) Johnston & Tiegs, 1922 to be the type species of the genus. *Protomicrocotyle mirabilis* had been collected from a fish, *Caranx hippos*, found in the New York Aquarium (MacCallum, 1918). However, although the study had many detailed descriptions (Johnston & Tiegs, 1922), it lacked precise morphological information for the subfamily-level taxon and for *P. mirabilis*.

Koratha (1955) provided more information on the species than Johnston & Tiegs (1922) and extended the distribution of *P. mirabilis* to Port Aransas, Texas, in the northwest Gulf of Mexico. In addition, he provided measurements and discussed the arguments by Sproston (1946) for moving the genus to Vallisiinae Price, 1943, Discocotylidae Price, 1936. Unnithan (1962) established the Superfamily Protomicrocotyloidea (Unnithan, 1962) for Protomicrocotylidae and placed *Protomicrocotyle*, along with several other genera, in the Protomicrocotylinae in a new family. Protomicrocotylidae currently is comprised of nine genera: *Abortipedia* Unnithan, 1963; *Bilaterocotyle* Chauhan, 1945; *Bilaterocotyloides* Ramalingam, 1961; *Chauhanocotyle* Khoche and Dad, 1975; *Lethacotyle* Manter & Price, 1953; *Neomicrocotyle* Ramalingam, 1960; *Protomicrocotyle* Johnston & Tiegs, 1922; *Vallisiopsis* Subhapradha, 1951; and *Youngiopsis* Lebedev, 1972 and approximately 42 species are currently assigned to the family.

*Protomicrocotyle mirabilis* has been reported from the Atlantic Ocean basin [as defined by Craig (2019)] from *C. hippos* from Ebrié Lagoon, Côte d'Ivoire, Western Africa; a description of that material was provided by Wahl (1972). The species also was reported from the Caribbean waters of Quintana Roo, Mexico by Bravo-Hollis (1989) who found it in *C. crysos*, *C. latus*, and *Caranx* sp.. *Protomicrocotyle mirabilis* has been reported several times in the Gulf of Mexico; Caballero y Caballero & Bravo-Hollis (1965) reported the species from *C. latus* collected from the waters off Tuxpan, Northern Veracruz; Bravo-Hollis (1989) reported the species in *C. hippos* and *Trachinotus carolinus*, collected from the Laguna de Sontecomapan and Las Cabañas, respectively, in Southern Veracruz, and Caballero y Caballero & Bravo-Hollis (1967) reported it from Campeche, Campeche, in *C. hippos*. *Protomicrocotyle ivoriensis* Wahl, 1972, the only other species known from the Atlantic Ocean basin, was described from specimens collected from *C. hippos* from Ebrié Lagoon, Côte d'Ivoire, Western Africa; *Protomicrocotyle manteri* Bravo-Hollis, 1966 and *Protomicrocotyle nayaritensis* Bravo-Hollis, 1979 have been described from the Pacific Coast of Mexico (Bravo-Hollis, 1966; Bravo-Hollis, 1979), and five other species have been described as parasites of fishes collected from the Pacific Ocean and the Indian Ocean basins (*Protomicrocotyle carangis*Pillai & Pillai, 1978, *Protomicrocotyle celebesensis*Yamaguti, 1953, *Protomicrocotyle madrasensis*Ramalingam, 1960, *Protomicrocotyle mannarensis*Ramalingam, 1960, and *Protomicrocotyle minutum* Ramalingam, 1960 (Yamaguti, 1953; Ramalingam, 1960; Pillai & Pillai, 1978).

In recent years, molecular approaches have been widely used for the exact identification of monogeneans and treatment of some unsolved taxonomical questions when only morphological methods were used (i.e. diagnosis, the discovery of cryptic species, delimitation of phenotypic variation, phylogeny) (Mendoza-Palmero et al., 2015; Tambireddy et al., 2016; Razo-Mendivil et al., 2016; Ondračková et al., 2020; Azizi et al., 2021; Ayadi et al., 2022). However, our present knowledge of molecular identification of the protomicrocotylids remains very limited due to the lack of genetic data. Currently, only four species of Protomicrocotylidae have been characterized with 28S, and two with cytochrome c oxidase subunit I (CO1) genes (Olson & Littlewood, 2002; Justine et al., 2013; Tambireddy et al., 2016) and genetic data for these species are available in GenBank® (Sayers et al., 2020) database. However, molecular data for many members of Mazocraeidea Bychowsky, 1937 are still lacking.

**Figure 1:**
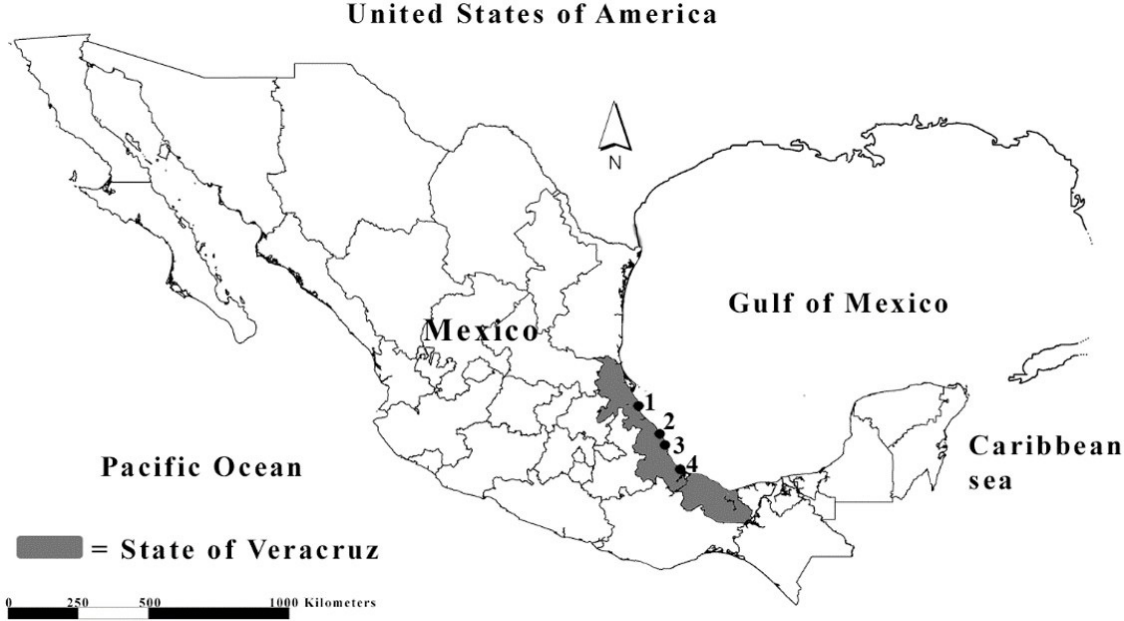
Geographic locations of the localities in the Coastal waters off Veracruz, Mexico, where fishes were collected: (1) Tuxpan; (2) Tecolutla; (3) Casitas; and (4) Puerto de Veracruz.

As part of the ongoing study of the helminths of marine fishes, individuals of *C. hippos*, and *C. latus* were collected from the coastal waters of Veracruz, Mexico (2005–2022) (Figure 1; Table 1) and necropsied for helminths. This study presents updated information on *P. mirabilis* [*sensu stricto*Kritsky et al. (2011)] and the description of a new species of *Protomicrocotyle*, a gill parasite of *C. latus* from Casitas and Puerto de Veracruz, Veracruz, Mexico. An integrative approach, including morphometrical categorization and molecular analyses of the cytochrome c oxidase subunit I and 28S rDNA was used to characterize these monogeneans.

**Table 1:** List of species of fish sampled in the Coastal waters off Veracruz, Mexico.

**Locality (number)**	**Species**	**Number of fish collected**	**Infected with *P. mirabilis***	**Infected with *P. veracruzensis* sp. nov.**	**Collection date**	**Coordinates**
Tuxpan (1)	*Caranx hippos*	4	4	‒	11 Mar 2010	20° 58' 09.2” N; 97° 19' 43.4 “W
		6	5	‒	12 Nov 2013	
		12	8	‒	19 Aug 2019	
Tecolutla (2)	*C. hippos*	1	‒	‒	27 May 2015	20° 29' 00.8” N; 97° 00' 07.2” W
		14	14	‒	12 Nov 2021	
Casitas (3)	*C. hippos*	22	18	‒	14 Nov 2021	20° 15' 31.5” N; 96° 47' 49.5” W
	*C. latus*	2	‒	1	4 May 2005	
		3	‒	‒	19 Aug 2019	
Puerto de Veracruz (4)	*C. hippos*	8	7	‒	24 May 2022	19° 13' 11.2” N; 96° 09' 24.4” W
	*C. latus*	1	‒	1	24 May 2022	

The number in the parenthesis is represented in Figure 1.

## Materials and Methods

### Collection localities

The present study was carried out using fish collected from four localities (port cities) with the following formal names: Tuxpan de Rodriguez Cano, Tecolutla, Casitas, and Veracruz; all in the state of Veracruz de Ignacio de la Llave (INEGI, 2023). To avoid confusion, they will be referred to using the commonly used names: Tuxpan, Tecolutla, Casitas, and Puerto de Veracruz, respectively. The state will be referred to simply as Veracruz or as the State of Veracruz.

### Specimen collection

During the period from 2005–2022, 73 specimens of *Caranx* were purchased from local fishermen, who caught them in littoral waters of the Gulf of Mexico, offshore of the four localities in the State of Veracruz, Mexico: 67 specimens of *C. hippos*, crevalle jack (jurel amarillo- Mexican common name) and six of *C. latus*, horse-eye jack (jurel blanco) (Figure 1; Table 1). The external body surface of each fish was examined for helminths using a magnifying glass and gill arches were excised, placed in a Petri dish with seawater, and examined using a stereomicroscope (Leica Zoom 2000). Members of *C. hippos* were found to be infected with *P. mirabilis* and members of *C. latus* were infected with an undescribed species of that genus (Table 1). Each fish was infected with only a single species of monogenean.

Monogeneans, dead at the time of collection, were removed from gill filaments and transferred temporarily to dishes containing seawater. When all worms had been collected, they were fixed with Alcohol-Formalin-Acetic Acid (AFA) at room temperature for at least 12 h and then transferred for storage to 70% ethyl alcohol for morphological studies [following Pulido-Flores & Monks (2005)]; other specimens were fixed and stored in 96-100% ethyl alcohol for molecular studies.

### Morphological and morphometric analysis

**Figure 2:**
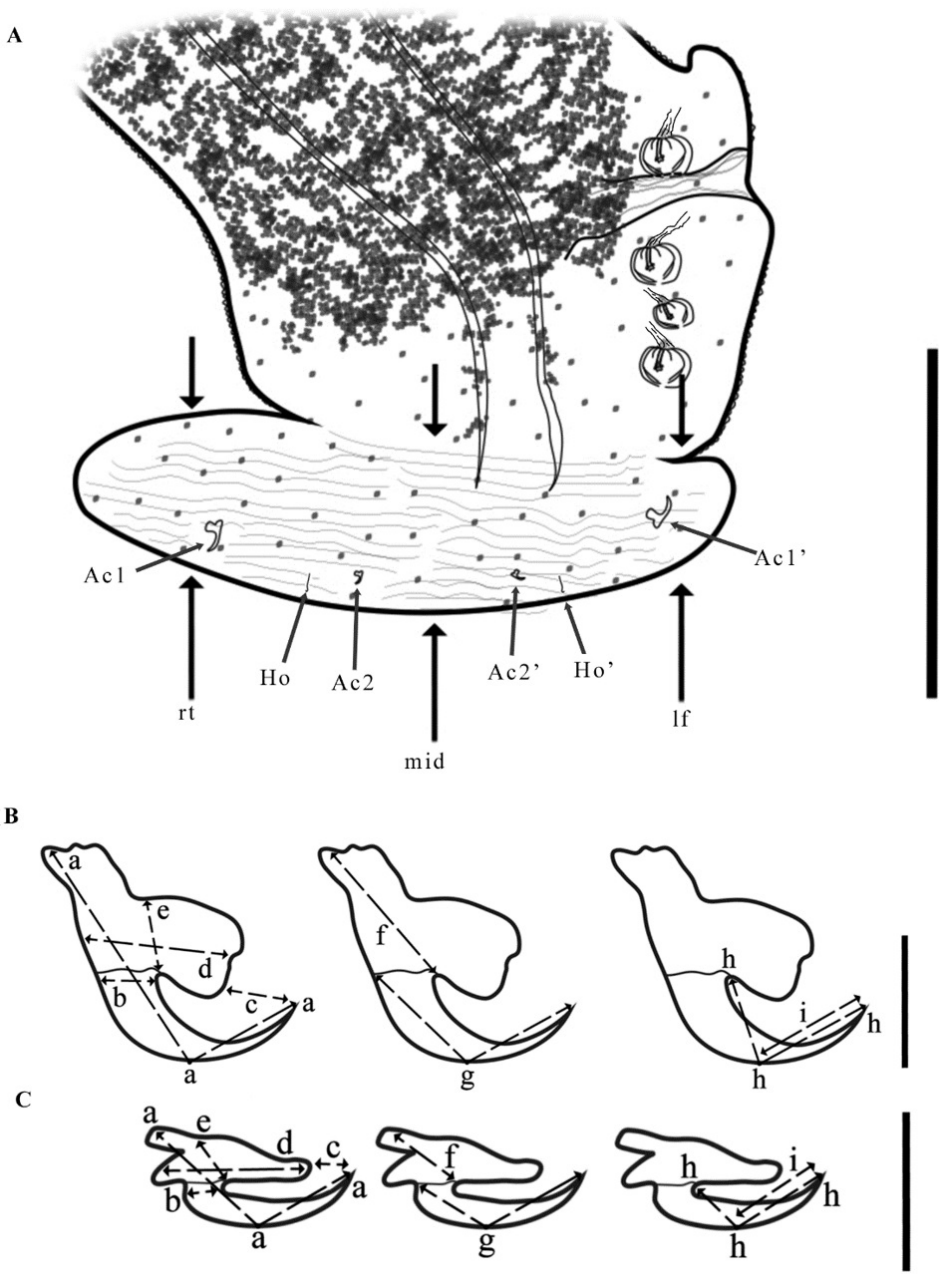
Scheme of measurements of the haptoral lappet and sclerotized structures. A. Haptoral lappet; rt = right side lateral haptoral lappet, length; mid = middle haptoral lappet length; lf = left side lateral haptoral lappet, length; Ac1 = right lateral anchor; Ac1’ = left lateral anchor; Ac2 = right medial anchor; Ac2’ = left medial anchor; Ho = right central hook; Ho’ = left central hook. Bars 350 µm. B. Lateral anchor, Bar 15 µm. C. Median anchor, Bar 17 µm; a = anchor, total length; b = anchor, width; c = opening; d, e = internal root (length and width, respectively); f = external root; g = length external blade; h = length internal blade; i = point.

Specimens were stained using Gomori’s trichrome, Mayer’s carmalum, or Delafield’s hematoxylin, dehydrated in an ethanol series, cleared in methyl salicylate, and mounted individually as whole-mounts on slides in Canada balsam. Morphometric comparisons were made using the information available in the literature for species of *Protomicrocotyle* reported in the Pacific and Atlantic Oceans and specimens borrowed from two collections (cited below). Specimens were examined using a compound optical microscope equipped with differential interference contrast (DIC) optics and drawings were made with the aid of a drawing tube. Measurements were made using an ocular micrometer; all measurements are given in micrometers as the mean followed in parentheses by the range and the number of structures (n) measured. For comparison with the shape of the haptoral lappet of other species, the length of the lappet was measured at three positions: right side lateral haptoral lappet (rt), opposite to the clamps; the middle of the lappet (mid); and the left side of the lappet (lf), the side closest to the clamps (Figure 2A).

Measurements of the anchors follow, in part, those taken by Mariniello et al. (2004), Amine & Euzet (2005), Vaughan & Christison (2012), and Řehulková et al. (2013): the total length and width of each anchor (a, b, respectively), length of the opening (c), the length and the width of the internal root (d, e, respectively), and the lengths of the external root (f), the length of the external blade (g), the length of the internal blade (h), and the length of the point (i) were taken (Figure 2B, 2C). Terminology of the clamp sclerites followed Boeger & Kritsky (1993) and Bouguerche et al. (2020). The shapes (forms) of structures were classified according to Clopton (2004). The infection parameters were calculated in accordance with Bautista-Hernández et al. (2015) and Bush et al. (1997). For comparative purposes, some type and voucher specimens from the Colección Nacional de Helmintos (CNHE) were examined: *Protomicrocotyle mirabilis* (CNHE-82; 111; 161; 162; 163; 164; 165; 166); *P. manteri* (CNHE-21; 134; 344; 345; 346; 347; 348; 3114; 3115) and *P. nayaritensis* (CNHE-158; 159). Specimens were deposited in the CNHE, Harold W. Manter Laboratory Collection (HWML), and the Colección de Helmintos (CHE), Universidad Autónoma del Estado de Hidalgo.

In order to corroborate preliminary assignment of specimens collected found infecting fish from the localities of Tuxpan, Tecolutla, Casitas, and Puerto de Veracruz, Veracruz to *P. mirabilis*, the measurements provided by Kritsky et al. (2011) and those of the specimens of this study were compared to determine if there was a significant difference using a two-sample independent Mann-Whitney test. This test can be used to determine if two independent groups are from the same group (MacFarland & Yates, 2016), this case, the same species.

For the morphometric analyses, 91 morphological variables from 150 specimens of *Protomicrocotyle* were analyzed (88 continuous, and three meristic). The measurements were obtained from specimens that had been identified previously as members of *P. mirabilis* (CHE: Ver-10-42; Ver-19-318; 317; 341; 348; 349; Ver-15-126; Ver-21-10; Ver-22-162; Ver-22-169: CNHE: 82; 111), *P. manteri* (CNHE: 346; 348; 3114; 3115), *P. nayaritensis* (CNHE: 158; 159), *Neomicrocotyle pacifica* (Meserve, 1938 Yamaguti, 1968) (CNHE: 371; 372; 3116) and the specimens of *P*. *mirabilis* and the new species collected as part of this study were used for the construction of a Principal Component Analysis (PCA) and Discriminant Analyses (DA). Individual values were transformed from micrometers to log for statistical analyses. The PCA was used to order and visualize possible clustering among the morphometric characters by evaluating the PCA with the objective of reducing the number of variables introduced (Palacio et al., 2020). The DA determines to what degree the analyzed variables, measurements of objects or individuals, best explain the attribution of the difference of the groups to which said objects or individuals belong (Torrado Fonseca & Berlanga Silvente, 2013; Palacio et al., 2020). The analyzes were performed using Past 4.06b (Hammer et al., 2001).

### Molecular study

Fish (*C. hippos*) that had been collected from the four localities in 2019 and 2022 (Table 1) were found to be infected with *P. mirabilis* [*sensu stricto* (Kritsky et al., 2011)]. An undescribed species was found to infect *C. latus* from Puerto de Veracruz. Specimens of *P. mirabilis* and the new species were prepared for molecular analyses. Genomic DNA was extracted using a DNeasy Blood and Tissue Kit (Qiagen, Hilden, Germany) following the manufacturer’s protocol. CO1 fragments were amplified using published primers JB3-F (ASmit1) (5’-TTTTTTGGGCATCCTGAGGTTTAT-3’) and JB4-R (ASmit2) (5’-TAAAGAAAGAACATAATGAAAATG-3’) (Bowles et al., 1993; Tambireddy et al., 2016), and the primers LSU5-F (5’-TAGGTCGACCCGCTGAAYTTAAGC-3’) and EC-D2-R (5’-CCTTGGTCCGTGTTTCAAGACGGG-3’) were used for 28S rDNA amplification (Littlewood et al., 1997; Tkach et al., 2003). The Polymerase Chain Reactions (PCR) were performed in a total of 25 µL, consisting of 4 µL of template DNA, 4.85 µL of master solution (0.15 µL of Taq DNA polymerase (5 µ/mL; BioTecMol), 1 µL dNTPs (2.5 mM; Promega), 0.2 µL of each primer (10 nM), 1.8 µL of 5x PCR buffer (BioTecMol), 1.5 µL of MgCl_2_ (25 mM; BioTecMol)), and 16.15 µL of distilled water.

The cycling conditions included initial denaturation at 94°C for 5 min, followed by 38 cycles of 94°C for 30 s, 45°C for 30 s, and 72°C for 1:10 min, and a final extension of 7:00 min at 72°C. PCR products were visualized in an electrophoresis agarose gel and were purified using polyethylene glycol (PEG) protocol and were sequenced using BigDye Terminator v3.1 Cycle Sequencing Kit in 10 µL reactions and 3730xl DNA Analyzer-Thermo Fisher Scientific using the JB3-F and LSU5-F primers, respectively, performed at the Instituto de Biología, UNAM, Mexico. Sequence data and electropherograms were inspected and edited using Pregap4 and Gap4 modules by Staden software V.1.6 (Staden, 1996).

Sequences obtained in the present study for CO1 and 28S regions were aligned with sequences from other protomicrocotylid retrieved from GenBank and *Allodiscocotyla diacanthi* Unnithan, 1962 (Allodiscocotylidae Tripathi, 1959 was used as the outgroup, based on the phylogenetic analysis by Tambireddy et al. (2016) (Table 2). Average *p-distance* between conspecific sequences from GenBank and collected samples (Table 2) were calculated in MEGA 11 (Tamura et al., 2021). A distance matrix was used for clustering analysis and the presentation of tree topology. The Neighbor-Joining (NJ) method was used to builds a tree from a matrix of pairwise evolutionary distances relating to the set of taxa being studied, therefore, the algorithm of the method finds the pairs of sequences that minimize the total length of the topology of the tree in each iteration (Gascuel & Steel, 2006; Saitou & Nei, 1987). The NJ analyses from CO1 and 28S was performed in MEGA 11 with bootstrap analysis based on 1000 resampling of each data set. The trees were edited in Adobe^®^ Photoshop^®^.

**Table 2:** List of monogeneans included in the genetic distances analyses and Unweighted Pair Group Method with Arithmetic Mean analyses, and GenBank accession numbers of sequences from the partial CO1 and 28S genes. New sequences obtained for the present study are in bold.

**Species of monogeneans**	**Host**	**GenBank ID**		**Reference for sequences**
		**CO1**	**28S**	
**Protomicrocotylidae**				
*Protomicrocotyle mirabilis*	*Caranx hippos*	**OR282821– OR282832**	**OR282885– OR282894**	Present study
*Protomicrocotyle veracruzensis* sp. nov.	*C*. *latus*	**OR282833– OR282836**	**OR282895– OR282898**	Present study
*Neomicrocotyle pacifica* (Meserve, 1938) Yamaguti, 1968	*C*. *hippos*	–	AF382043	Olson & Littlewood (2002)
*Neomicrocotyle* sp.	*C. sexfasciatus*	–	KF378589	Justine et al. (2013)
*Lethacotyle vera* Justine, Rahmouni, Gey, Schoelinck and Hoberg, 2013*	*C. papuensis*	–	KF378588	Justine et al. (2013)
*Bilaterocotyloides carangis* Ramalingam, 1961	*Megalaspis cordyla*	KF804043	–	Tambireddy et al. (2016)
		–	KJ201022	Tambireddy et al. (2016)
*Bilaterocotyloides madrasensis* Radha, 1966	*M*. *cordyla*	KF804041	KF804029	Tambireddy et al. (2016)
		–	KF804037	Tambireddy et al. (2016)
**Outgroup**				
**Allodiscocotylidae**				
*Allodiscocotyla diacanthi* Unnithan, 1962	*Scomberoides commersonnianus*	KF804045	KF804033	Tambireddy et al. (2016)
		KF804046	KF804038	Tambireddy et al. (2016)

* Reported as *Lethacotyle* sp. in GenBank.

## Results

### Morphology study

Class Monogenea van Beneden, 1858

Subclass Polypisthocotylea Odhner, 1912

Order Mazocraeidea Bychowsky, 1937

Family Protomicrocotylidae Johnston & Tiegs, 1922

Genus *Protomicrocotyle* Johnston & Tiegs, 1922

### *Protomicrocotyle mirabilis* (MacCallum, 1918) Johnston & Tiegs, 1922

*Description*. Based on 81 specimens, stained, and mounted to be viewed from ventral side of the body.

**Figure 3:**
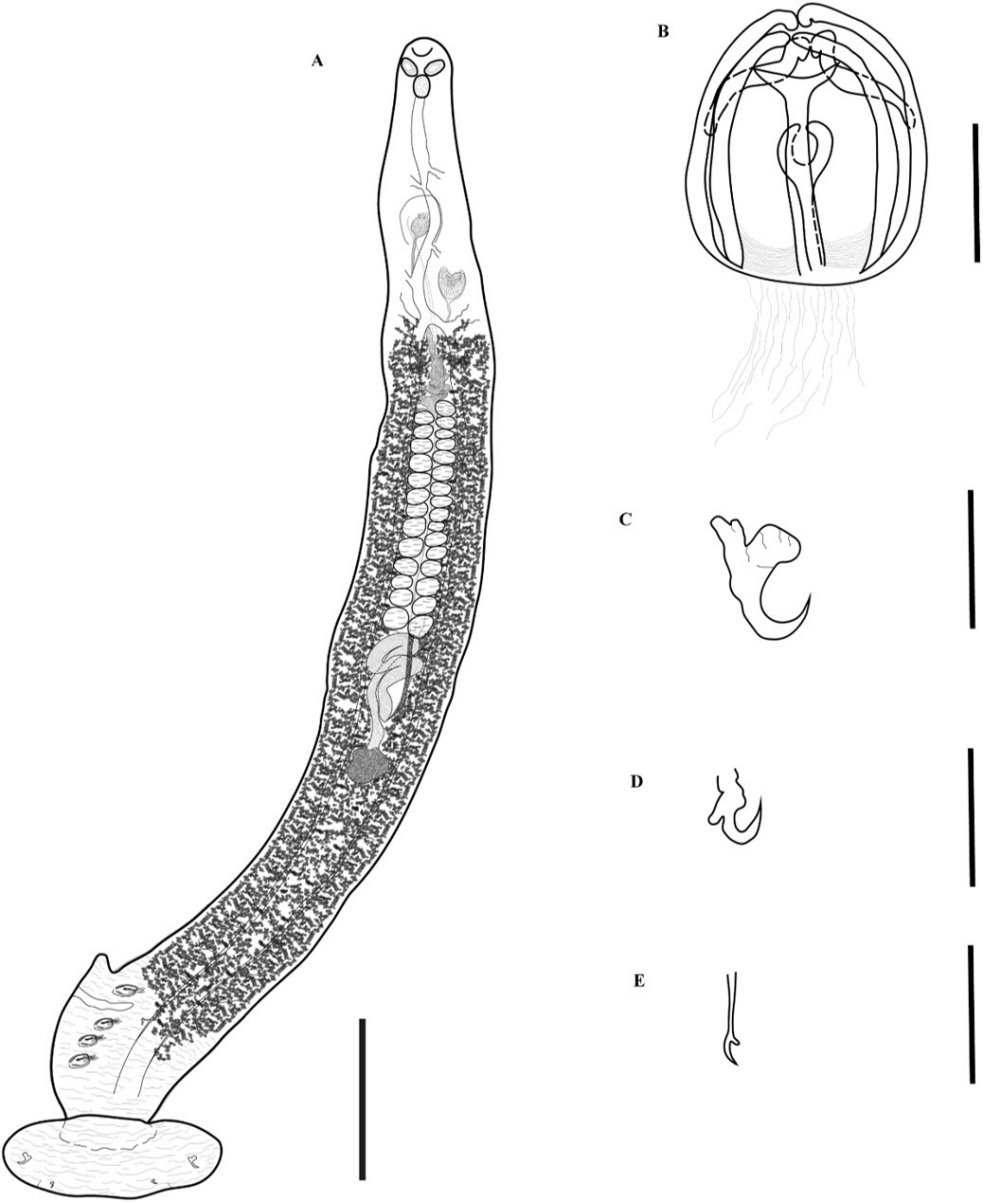
*Protomicrocotyle mirabilis*. A. Whole worm (ventral view), Bar 500 µm. B. Details of clamp, Bar 32 µm. C. Lateral anchor, Bar 32 µm. D. Median anchor, Bar 32 µm. E. Hook, Bar 32 µm.

*Body* (Figure 3A), Table 3. Body fusiform [see Clopton (2004) for names of shapes], 2861 (805‒4673, n = 79) long (including haptoral lappet) and 295 (134‒537, n = 80) wide at level of middle of testicular field. Tegument with annular ridges in the region media of the body and posterior to the ovary that extend to posterior region of body, these striations on the margin of the body give it a serrated appearance (Figure 3A). Prohaptoral suckers anterolateral, muscular, oval; right oral sucker 44 (13‒68, n = 77) long, 31 (10‒48, n = 77) wide, and left sucker 44 (14‒67, n = 77) long, 31 (11‒48, n = 77) wide. Buccal cavity subterminal, ventral, ovoid-broadly, 26 (11‒52, n = 70) long, 31 (12‒67, n = 73) wide. Pharynx, posterior to buccal cavity, muscular, broadly elliptoid, 42 (14‒64, n = 77) long, 35 (14‒52, n = 77) wide. Esophagus long, with lateral diverticula, 495 (295–670, n = 66) long (Figure 3A). Cecal bifurcation posterior to male copulatory organ (MCO), 606 (380‒775, n = 66) from anterior end of body. Intestinal caecum lateral to midline and reproductive organs, extending into haptor; caecum with numerous short and lateral ramifications (Figure 3A).

**Table 3:** Morphometric comparison of different records of *Protomicrocotyle mirabilis* (taxonomic identity corroborated) and *P*. cf. *mirabilis* (taxonomic identity not corroborated). Measurements are presented in micrometers.

Species	***Protomicrocotyle mirabilis***	***P. mirabilis***	***P. mirabilis***
Locality	Veracruz (this study)	Florida Bay, Everglades National Park, Florida	New York Aquarium, USA
Host	***C. hippos***	***Caranx hippos***	***C. hippos***
Site	Gill filaments	Gill lamellae	Gill lamellae
	µm	µm	µm
Total length	2861 (805–4673, n =79)	2240 (1470–3080)	**3128**
Total width	295 (134–537, n =80)	322 (214–449)	200
Mouth length	26 (11–52, n =70)	16	‒
Mouth width	31 (12–67, n =73)	25	‒
Cecal bifurcation to anterior end	606 (380–775, n =66)	**346**	‒
Pharynx length	40 (14–64, n =77)	32	‒
Pharynx width	35 (14–52, n =77)	47 (39–57)	‒
Prohaptoral sucker length (right)	44 (13–68, n =77)	**23**	**31**
Prohaptoral sucker width (right)	31 (10–48, n =77)	**32**	**22**
Prohaptoral sucker length (left)	44 (14–67, n =77)	**24**	**30**
Prohaptoral sucker width (left)	31 (11–48, n =77)	**33**	**17**
Clamp length	45.7 (14.2‒65, n =71 )*	57 (49–67)	**55**
Haptor length	477 (165–815, n =76)	**338**	
Haptor width	255 (70–425, n =76)	**161**	**110**
Right side of haptoral lappet length	117 (60–217, n =72)	**64**	**73**
Left side of haptoral lappet length	120 (40–202, n =73)	**72**	**61**
Central haptoral lappet length	125 (40–214, n =74)	**88**	**134**
Haptoral lappet width	437 (105–785, n =75)	241	330
Lateral anchor length	41.5 (22.5‒65, n = 153)*	33 (29–37)	‒
Lateral anchor width	5 (2‒7.5, n = 153)*	**3**	‒
Medial anchor length	27.5 (9–37, n = 151)*	21 (19–22)	‒
Medial anchor width	4 (2–6, n = 151)*	**2**	‒
Hook length	17 (7–23, n = 144)*	18 (16–19)	‒
Hook width	3 (1–3, n = 138)*	**2**	‒
Number of testes	31 (21–38, n =77)	27 (23–33)	14–35
Testes length	45 (17–79, n =81)	43 (28–54)	**34.6**
Testes width	50 (14–86, n =80)	43 (28–54)	**49.3**
Testes-Ratio length: wide	1:1.1	1:1	1:1.4
Male copulatory organ length	54 (22–82, n =80)	**77**	‒
Male copulatory organ width	41 (11–66, n =80)	50 (42–56)	‒
Genital pore to anterior end distance	370 (140–660, n =81)	250	**452**
Vaginal vestibule length	69 (28–94, n =80)	82 (66–99)	**52.5**
vaginal vestibule width	51 (19–77, n =80)	59 (41–75)	**48**
Vaginal opening to anterior end	464 (175–740, n =80)	**330**	**567**
Vaginal opening to genital pore	112 (40–180, n =80)	**88**	**134**
Egg length	157 (88–206, n =41)	180 (167–193)	**183**
Egg width	49 (24–68, n =41)	68 (66–70)	**85.5**
Number of spines in male copulatory organ (MCO)	22 (16–29, n =71)	19 (16–21)	**20**
Number of spines in vaginal vestibule	45 (31–65, n =63)	**49**	‒
Reference	Present study	Kritsky et al. (2011)	MacCallum (1918)

Species	***P. mirabilis***	***P.* cf. *mirabilis***	***P.* cf. *mirabilis***
Locality	Tuxpan, Veracruz	Sontecomapan, Veracruz, Mexico	Las Cabanas, Veracruz, Mexico
Host	***C. latus***	***C. hippos***	***C. hippos***
Site	Gill lamellae	Gill lamellae	Gill lamellae
	µm	µm	µm
Total length	1254‒2451	1482‒3706	1881‒2052
Total width	149‒387	171‒399	285‒342
Mouth length	**19**	‒	‒
Mouth width	**44**	‒	‒
Cecal bifurcation to anterior end	**375**	**‒**	**‒**
Pharynx length	**43**	‒	‒
Pharynx width	**44**	‒	‒
Prohaptoral sucker length (right)	**44**	**‒**	**‒**
Prohaptoral sucker width (right)	**29**	**‒**	**‒**
Prohaptoral sucker length (left)	**44**	**‒**	**‒**
Prohaptoral sucker width (left)	**27**	**‒**	**‒**
Clamp length	48‒52	‒	‒
Haptor length	**342**	**‒**	**‒**
Haptor width	149‒298	**‒**	**‒**
Right side of haptoral lappet length	**49**	**‒**	**‒**
Left side of haptoral lappet length	**44**	**‒**	**‒**
Central haptoral lappet length	**71**	**‒**	**‒**
Haptoral lappet width	283	**‒**	**‒**
Lateral anchor length	‒	‒	‒
Lateral anchor width	**‒**	**‒**	**‒**
Medial anchor length	‒	‒	‒
Medial anchor width	**‒**	**‒**	**‒**
Hook length	‒	‒	‒
Hook width	**‒**	**‒**	**‒**
Number of testes	20‒30	18‒29	17‒26
Testes length	37‒41	‒	‒
Testes width	33‒59	‒	‒
Testes-Ratio length: wide	1:0.8	‒	‒
Male copulatory organ length	**49**	**‒**	**‒**
Male copulatory organ width	**38**	**‒**	**‒**
Genital pore to anterior end distance	231	**‒**	**‒**
Vaginal vestibule length	**88**	**‒**	**‒**
vaginal vestibule width	**60**	**‒**	**‒**
Vaginal opening to anterior end	268‒402	**‒**	**‒**
Vaginal opening to genital pore	**77**	**‒**	**‒**
Egg length	‒	‒	‒
Egg width	‒	‒	‒
Number of spines in male copulatory organ (MCO)	18‒20	17‒19	16‒17
Number of spines in vaginal vestibule	**‒**	**‒**	**‒**
Reference	Caballero y Caballero & Bravo-Hollis (1965)	Bravo-Hollis (1989)	Bravo-Hollis (1989)

Species	***P.* cf. *mirabilis***	***P.* cf. *mirabilis***	***P.* cf. *mirabilis***	***P.* cf. *mirabilis***	***P.* cf. *mirabilis***
Locality	Chetumal, Quintana Roo, Mexico	Isla Cozumel, Quintana Roo, Mexico	Isla Mujeres, Quintana Roo, Mexico	Port Aransas, Texas, USA	Ebrié Lagon, near Abidjan
Host	***C. latus***	***Caranx* sp.**	***C. crysos***	***C. hippos***	***C. hippos***
Site	Gill lamellae	Gill lamellae	Gill lamellae	Gill lamellae	Gill lamellae
				µm	µm
Total length	1938‒3192	2565‒3078	2223‒2280	5400	2000‒2900
Total width	171‒285	228‒399	470‒620	275	250‒550
Mouth length	‒	‒	‒	‒	‒
Mouth width	‒	‒	‒	‒	‒
Cecal bifurcation to anterior end	**‒**	**‒**	**‒**	‒	**345**
Pharynx length	‒	‒	‒	‒	‒
Pharynx width	‒	‒	‒	‒	‒
Prohaptoral sucker length (right)	**‒**	**‒**	**‒**	‒	**‒**
Prohaptoral sucker width (right)	**‒**	**‒**	**‒**	‒	**‒**
Prohaptoral sucker length (left)	**‒**	**‒**	**‒**	‒	**‒**
Prohaptoral sucker width (left)	**‒**	**‒**	**‒**	‒	**‒**
Clamp length	‒	‒	‒	60	60
Haptor length	**‒**	**‒**	**‒**	‒	**138**
Haptor width	**‒**	**‒**	**‒**	‒	**‒**
Right side of haptoral lappet length	**‒**	**‒**	**‒**	‒	**166**
Left side of haptoral lappet length	**‒**	**‒**	**‒**	‒	**178**
Central haptoral lappet length	**‒**	**‒**	**‒**	‒	**154**
Haptoral lappet width	**‒**	**‒**	**‒**	200	**416**
Lateral anchor length	‒	‒	‒	‒	‒
Lateral anchor width	**‒**	**‒**	**‒**	‒	**‒**
Medial anchor length	‒	‒	‒	‒	‒
Medial anchor width	**‒**	**‒**	**‒**	‒	**‒**
Hook length	‒	‒	‒	‒	‒
Hook width	**‒**	**‒**	**‒**	‒	**‒**
Number of testes	20‒25	20‒21	24‒25	‒	28‒32
Testes length	‒	‒	‒	‒	75‒85
Testes width	‒	‒	‒	‒	45‒55
Testes-Ratio length: wide	‒	‒	‒	‒	1:0.6
Male copulatory organ length	**‒**	**‒**	**‒**	‒	**107**
Male copulatory organ width	**‒**	**‒**	**‒**	‒	**71**
Genital pore to anterior end distance	**‒**	**‒**	**‒**	‒	**309**
Vaginal vestibule length	**‒**	**‒**	**‒**	‒	**71**
vaginal vestibule width	**‒**	**‒**	**‒**	‒	**95**
Vaginal opening to anterior end	**‒**	**‒**	**‒**	‒	**300**
Vaginal opening to genital pore	**‒**	**‒**	**‒**	‒	**‒**
Egg length	‒	‒	‒	‒	‒
Egg width	‒	‒	‒	‒	‒
Number of spines in male copulatory organ (MCO)	18‒19	16‒20	18‒19	‒	15‒20
Number of spines in vaginal vestibule	**‒**	**‒**	**‒**	‒	**‒**
Reference	Bravo-Hollis (1989)	Bravo-Hollis (1989)	Bravo-Hollis (1989)	Koratha (1955)	Wahl (1972)

* Average of all anatomical structures measured.

Values in bold were taken of the line drawings.

*Haptor* (Figure 3A). Haptor asymmetrical, comprised of four lateral clamps on the left side and a terminal haptoral lappet; (no asymmetry of these structures observed); 477 (165‒815, n = 76) long, 255 (70‒425, n = 76) wide at the level of the haptoral groove. Clamps located ventrally, each with a short muscle peduncle, positioned on left side of worm (on right side of drawing of worm in ventral view) (Figure 3B). First clamp (anterior to haptoral groove) 46 (12‒66, n = 65) long, 35 (11‒52, n = 65) wide; second clamp 46 (14‒67, n = 71) long, 37 (13‒73, n = 71) wide; third clamp 46 (12‒65, n = 74) long, 37 (13‒56, n = 74) wide; fourth clamp 46 (19‒66, n = 72) long, 36 (12‒54, n = 72) wide. Clamps typical of Family Gastrocotylidae. Small haptoral groove, with raised edges, between the first and second clamps (Figure 3A). Haptoral lappet elongate ovate, wider than long. Length of lappet measured in three regions (Figure 2A); right side of haptoral lappet (rt) 117 (60‒217, n = 72) long, middle region of haptoral lappet (mid) 125 (40‒214, n = 74) long, left side of haptoral lappet (lf) 120 (40‒202, n = 73) long. Haptoral lappet 437 (105‒785, n = 75) wide, armed with two pairs of anchors, and one pair of hooks (Figure 3C, 3D, 3E).

**Figure 4:**
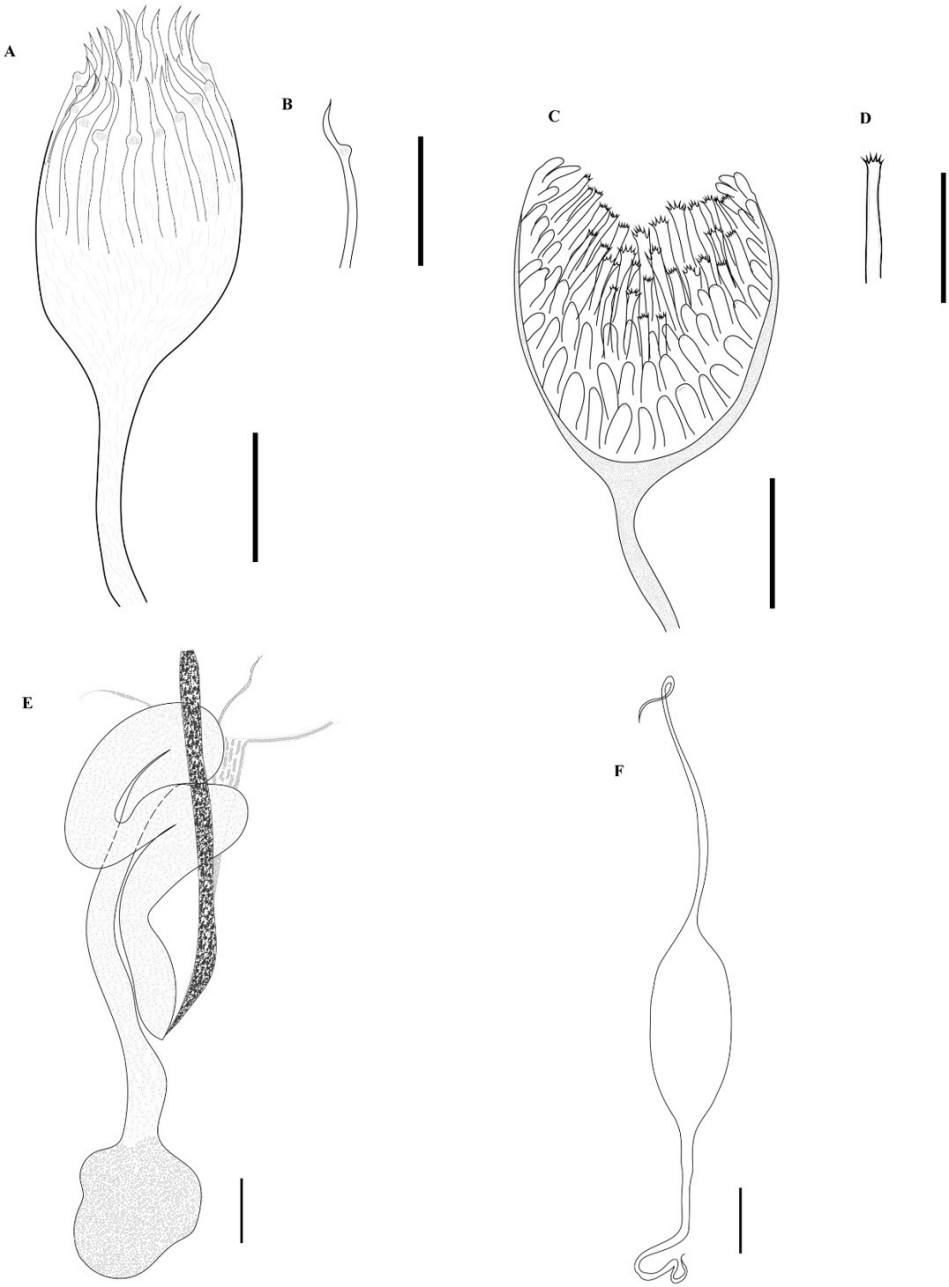
Female and male reproductive organs of *Protomicrocotyle mirabilis*. A. Male copulatory organ, Bar 32 µm. B. Spine of male copulatory organ, Bar 32 µm. C. Vaginal vestibule, Bar 32 µm. D. Spine of vaginal vestibule, Bar 32µm. E. Female reproductive organs, Bar 50 µm. F. Egg, Bar 50 µm.

*Male reproductive structures* (Figures 3A, 4A). Testes 31 (21‒38, n = 77) in number, very broadly elliptoid, intercecal, anterior to descending ducts of germarium, arranged in two parallel fields, each field with one regular column of testes. Testes 45 (17‒79, n = 81) long, 50 (14‒86, n = 80) wide. Length to width ratio L:W= 1:1.1 (Figure 3A). Vas deferens dorsal to testes, running in zigzag pattern from anterior part of testes to MCO. Male copulatory organ 54 (22‒82, n = 80) long, 41 (11‒66, n = 80) wide, subspherical, armed with 22 (16‒29, n = 71) spines (Figure 4A). Spines of MCO hooklike, 36 (16‒50, n = 78) long, 3 (2‒6, n = 78) wide, arranged in a circle on anterior part of MCO (Figure 4B). Genital atrium ventral, near midline, anterior to cecal bifurcation, opening at 370 (140‒660, n = 81) from anterior end of body.

*Female reproductive structures* (Figure 4C, 4E). Germarium intercecal, post-testicular, comprised of germarial bulb with immature oöcytes, 116 (36‒199, n = 75) long, 69 (17‒124, n = 75) wide, with a wide ascending duct and an irregularly descending duct that form loops that contain mature oöcytes. The oviduct ends in the oötype; uterus ascends from oötype to the genital atrium (Figure 4E). Vaginal pore ventral, anterior to vaginal vestibule. Vaginal vestibule 69 (28‒94, n = 80) long, 51 (19‒77, n = 80) wide, located 464 (175‒740, n = 80) from anterior end of body and 112 (40‒180, n = 80) from the genital pore, lateral to midline on the side opposite to that having the haptoral clamps (Figure 4C). Vaginal vestibule armed with approximately 45 (31‒65, n = 63) spines 34 (13‒50, n = 76) long, 3 (1‒5, n = 76) wide; the spines of the anterior region of the vestibule are larger and those of the middle and basal region smaller. These spines have small spine-like extensions on the distal region of each spine (Figure 4D). The vaginal duct descends from the vaginal vestibule to the germarium and connects to the oötype in the ventral region of the germarium between descending duct and germarium bulb. Seminal receptacle not observed. Vitelline glands in two lateral fields, starting just posterior to vaginal vestibule, overlapping ceca anteriorly and posteriorly and the lateral margin of the testes, uniting posterior to germarium, extending to posterior region of body but not reaching the haptoral lappet (Figure 3A).

*Eggs* (Figure 4F). Uterus contains 2 (1‒3, n = 41) eggs. Eggs elongate, elliptoid, with long polar filaments at each pole; length, not including polar filaments, 157 (88‒206, n = 41), 49 (24‒68, n = 41) wide. Filament in anterior end (as positioned in uterus) 217 (49‒308, n = 19) long, 5(2‒8, n = 19) wide, and posterior end 187 (84‒317, n = 26) long, 5 (4‒8, n = 26) wide.

### Taxonomic summary

**Type Host:***Caranx hippos* (Carangidae).

**Common name:** crevalle jack.

**Additional hosts:***Caranx latus* (Carangidae) (Caballero y Caballero & Bravo-Hollis, 1965).

**Type locality:** New York Aquarium, New York (MacCallum, 1918).

**Additional localities:** Everglades National Park, Florida (Kritsky et al., 2011); Littoral waters off the Gulf of Mexico off Tuxpan, Tecolutla, Casitas and Puerto de Veracruz, Veracruz, Mexico (this study) (Figure 1; Table 1).

**Site of infection:** Gill filaments.

**Abundance, prevalence, mean intensity of infection and range of intensity:** General infection characterization: *C. hippos*, 7.21 of abundance, 56 infected fish of 67 examined (83.58%), 8.63 and 1‒65. Tuxpan: 10.64 of abundance, 17 infected fish of 22 examined (77.27%), 13.76 and 3‒44; Tecolutla: 11 of abundance, 14 infected fish of 15 examined (93.33%), 11.79 and 4‒59; Casitas: 13.27 of abundance, 18 infected fish of 22 examined (81.82%), 16.22 and 1‒65, and; Puerto de Veracruz: 14.25 of abundance, 7 infected fish of 8 examined (87.50%), 16.29 and 2‒53.

**Specimens deposited:** vouchers CNHE-12822 to 12825, HWML-216985 to 216994, CHE-P00147.

**Genbank accession numbers:** Cytochrome C Oxidase Subunit I (CO1) OR282821 to OR282832; 28S rDNA OR282885 to OR282894

### Remarks

Members of *Protomicrocotyle* can be characterized by a suite of characters that include: having an asymmetrical haptor with four Gastrocotylidae-type clamps in a longitudinal row on the side opposite to the vaginal vestibule; clamps with accessory sclerites; the haptoral lappet is transversely elongate (wider than long), armed with two pairs of anchors (larger anchors positioned laterally with smaller anchors between the larger ones), and one pair of hooks (located between the smaller paired anchors); the esophagus and ceca have diverticula; vitellaria and ceca may or may not extend into the haptor; the testes are relatively numerous, with variations in shape, size, and arrangement, but always are anterior to the female complex; the MCO bulbous, may be muscular, and is provided with a crown of numerous spines; genital pore ventral to esophagus; ovary consisting of a germarium and a tubular duct, winding or not, post-testicular; genitointestinal canal present, crossing ovary or not; eggs with filament at each pole; vagina opening ventrally to the right or left, posterior to genital pore, armed or not, with numerous spines of different shapes; vitellaria extends lateral and dorsal to ceca (Yamaguti, 1963). They are parasites of marine teleost’s, principally of fishes of Carangidae (Yamaguti, 1963). The material described herein has the diagnostic morphological characteristics of this genus. The reason for reporting this information in detail is the necessity to corroborate the identity of the specimens collected from Tuxpan so that the molecular data could be linked to *P. mirabilis*, so it could be compared to that of the new species.

The results of the Mann-Whitney test indicated that there are no significant differences [*Z (U)* = 0.729; *P* = 0.465] in the measurements between the specimens of *Protomicrocotyle mirabilis* from Florida, as described by Kritsky et al. (2011), and those specimens collected from Veracruz (Figure 1; Table 1). In accordance with the redescription by Kritsky et al. (2011) and the specimens collected as part of this study, *P. mirabilis* is characterized by having the characters of the genus and of the species, as detailed above. Measurements for *P. mirabilis* presented in this work are shown in comparison with that reported by Kritsky et al. (2011) in Table 3. Comparing the measurements in Table 3, it is possible to observe variation with respect to some variables, but as demonstrated by the Mann-Whitney test, there are no significant differences. However, it is worth mentioning that there are some characters that are outside the ranges established by Kritsky et al. (2011) in their redescription of *P. mirabilis*, such as body length, average testes size, the number of spines in the male reproductive organ, the vaginal vestibule, and the number of testes, among other variables (Table 3) of the specimens in this study are larger than what was mentioned by Kritsky et al. (2011). Therefore, it is necessary to carry out studies of specimens from other locations within the different biogeographical provinces that make up the Gulf of Mexico (Carolina Province and Caribbean Province) (Briggs & Bowen, 2012) and of the different ecoregions of which these provinces are pertain (Lara-Lara et al., 2008; Mendelssohn et al., 2017) that include the analysis of morphological and molecular characteristics. In this manner, existing variation in morphology can be attributed to intraspecific variation or if it reveals a complex of cryptic species. However, the current information is interpreted as a confirmation that *P. mirabilis* (*sensu stricto*Kritsky et al. (2011)) is widely distributed within the Gulf of Mexico. Verified locality reports include Tuxpan [this study and Caballero y Caballero & Bravo-Hollis (1965)], Tecolutla (this study), Casitas (this study), Puerto de Veracruz (this study) and Campeche [this study and Caballero y Caballero & Bravo-Hollis (1967)]. Previous records from other localities in the Gulf of Mexico (Bravo-Hollis, 1989; Mendoza-Garfias et al., 2017; Montoya-Mendoza et al., 2017), Caribbean Sea (Bravo-Hollis, 1989), South America (Boada et al., 2012; Vianna et al., 2020) and the Republic of Côte d'Ivoire (Wahl, 1972) could not be corroborated from existing material. Additional material that includes specimens for comparative morphological and molecular analyses must be collect in order to establish the limits of the distribution of *P. mirabilis*.

### *Protomicrocotyle veracruzensis* sp. nov.

**Figure 5:**
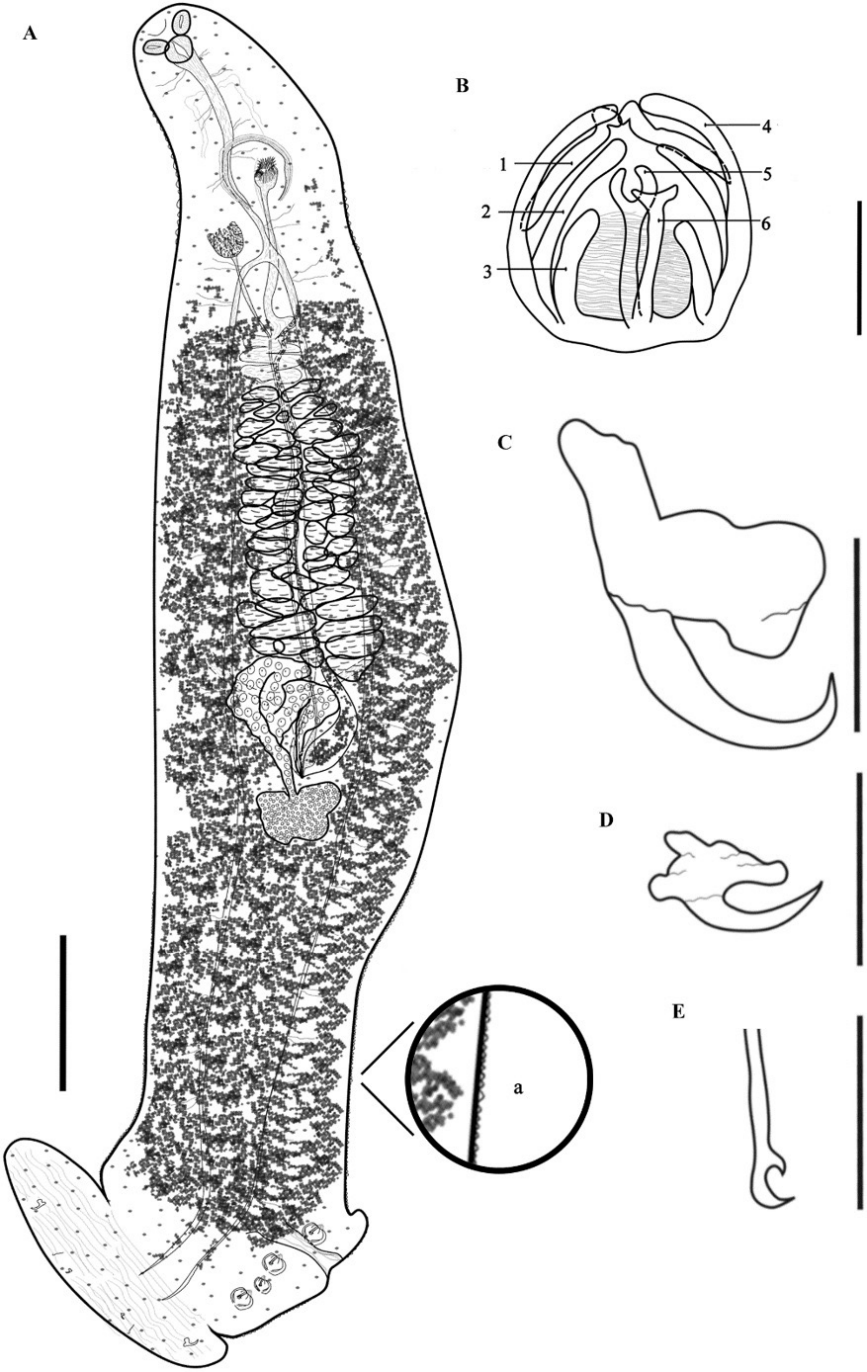
*Protomicrocotyle veracruzensis* sp. nov. (holotype). A. Whole worm (ventral side up), a **=** inset showing marginal folds, Bar 500 µm. B. Details of clamp, 1 = oblique sclerite; 2 = dorsal jaw;, 3 = dorsal arm of the ventral jaw; 4 = ventral jaw; 5 = accessory skeletal piece; 6 = ventral arm of median spring, Bar 30 µm. C. Lateral anchor, Bar 15 µm. D. Median anchor, Bar 20 µm. E. Hook, Bar 15 µm.

**Figure 6:**
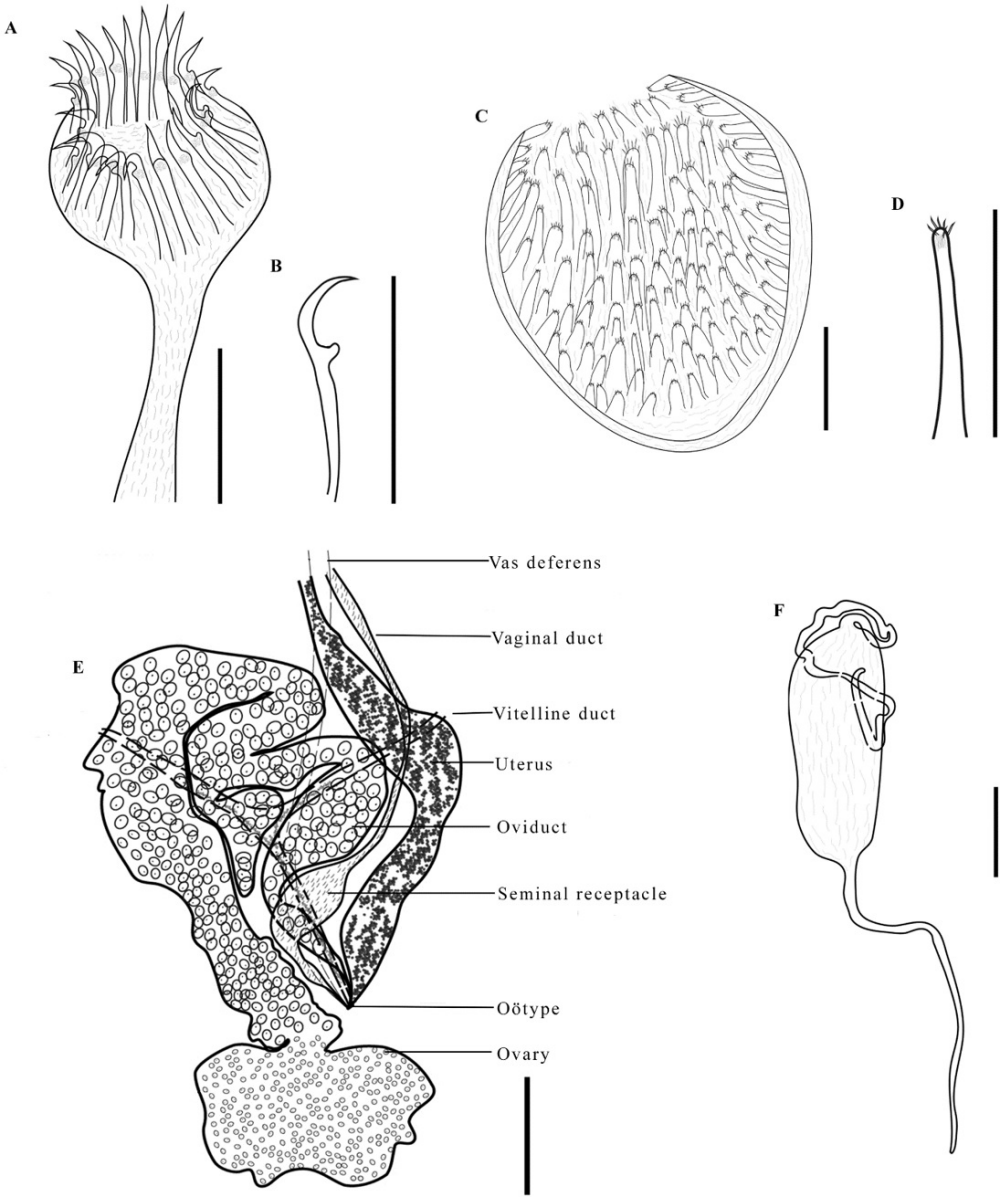
*Protomicrocotyle veracruzensis* sp. nov. A. Male copulatory organ, Bar 50 µm. B. Spine of male copulatory organ, Bar 40 µm. C. Vaginal vestibule, Bar 25 µm. D. Spine of vaginal vestibule, Bar 40 µm. E. Female reproductive organs, Bar 200 µm. F. Egg, Bar 80 µm.

**Figure 7:**
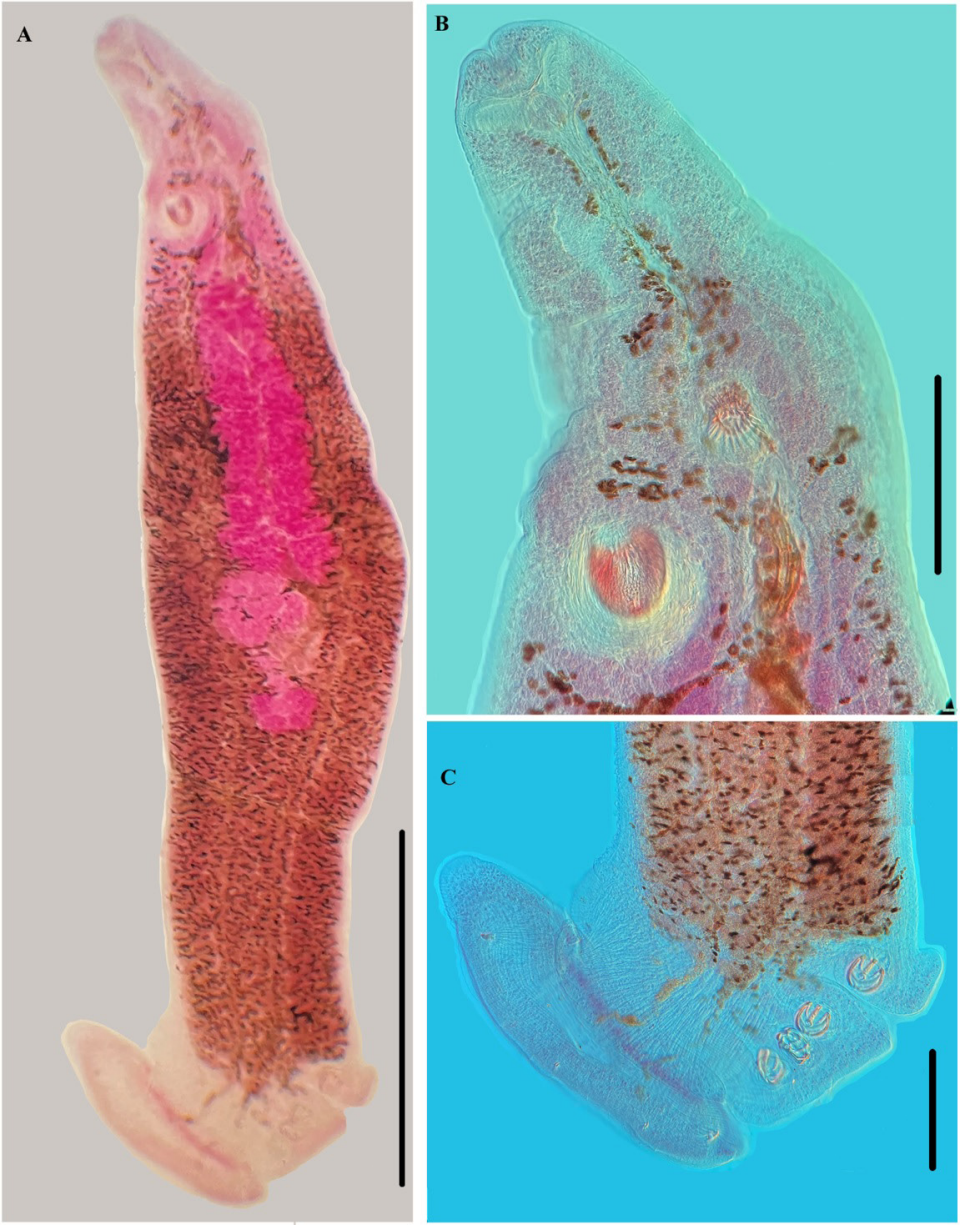
Photograph of *Protomicrocotyle veracruzensis* sp. nov. (Holotype). A. Whole specimen (ventral view), Bar 125 µm. B. Prohaptor, Bar 500 µm. C. Haptor, Bar 500 µm.

*Description* (Figures 2, 5, 6, 7; Tables 4, 5). Based on 24 adult specimens, stained, and mounted to be viewed from ventral side of the body. Measurements and data of other species are presented in Table 4.

**Table 4:** Comparative morphological characteristics of the species of the genus *Protomicrocotyle*. Measurements are presented in micrometers.

Species	***Protomicrocotyle veracruzensis* sp. nov.**	***P. mirabilis*** (redescription)
Hosts	*Caranx latus*	*C. hippos* and *C. latus*
Location	Casitas and Puerto de Veracruz, Veracruz, México	Florida, EUA and Veracruz, Mexico
Total length	3858 (3209‒4709)	805‒4673
Total Width	804 (586‒1098)	134‒537
Haptor width	575 (415‒980)	70‒425
Right side of haptoral lappet length	173 (136‒240)	60‒217
Left side of haptoral lappet length	194 (124‒259)	40‒202
Central haptoral lappet length	206 (164‒259)	40‒214
Haptoral lappet width	769 (580‒920)	105‒785
Clamps length	64 (47‒74)*	14.2‒67
Number of testes	47 (36‒69)	14‒38
Testes length	47 (31‒59)	17‒79
Testes width	142 (96‒176)	14‒86
Testes-Ratio length: width	01:03	1:0.8‒1:1.1
Vaginal opening to anterior end	610 (440‒730)	175‒740
Vaginal opening to genital pore	506 (340‒615)	140‒660
Number of spines in MCO	23 (16‒28)	16‒29
Number of spines in vestibule vaginal	49 (33‒59)	31‒65
Reference	Present study	Present study

Species	***P. manteri***	***P. nayaritensis***	***P. ivoriensis***
Hosts	*Trachinotus paitensis*	*C. caninus*	*C. hippos*
Location	La Paz, BCS, Mexico	Nayarit, Mexico	Ivory Coast
Total length	1360‒3680	6820‒8580	3200‒4600
Total Width	400‒800	770‒1990	600‒1200
Haptor width	**590**	1050	442
Right side of haptoral lappet length	**136**	**250**	**173**
Left side of haptoral lappet length	**121**	**150**	**178**
Central haptoral lappet length	**181**	**300**	**134**
Haptoral lappet width	**348**	643‒801	500
Clamps length	50‒55	45‒48	44
Number of testes	22‒33	46‒48	50
Testes length	60.2	88	58.8
Testes width	116.5	129	99.5
Testes-Ratio length: width	01:01.8	01:01.4	01:01.6
Vaginal opening to anterior end	84‒155	1072‒1258	442
Vaginal opening to genital pore	289‒471	858‒1001	365
Number of spines in MCO	33‒38	48‒54	25‒26
Number of spines in vestibule vaginal	15	22‒46	‒
Reference	Bravo-Hollis (1966)	Bravo-Hollis (1979)	Wahl (1972)

Species	***P. celebensis***	***P. madrasensis***	***P. mannarensis***
Hosts	*Caranx* sp.	*C. affinis*	*C. sexfasciatus*
Location	Indonesia	India	India
Total length	4000‒5100	2390	2880‒3640
Total Width	600‒830	410	480‒1140
Haptor width	‒	300	434
Right side of haptoral lappet length	**111**	**250**	**130**
Left side of haptoral lappet length	**111**	**250**	**152**
Central haptoral lappet length	**166**	**112**	**213**
Happtoral lappet width	‒	240	320‒440
Clamps length	‒	32‒48	30‒46
Number of testes	40‒55	64	42‒52
Testes length	‒	33‒38	21‒33
Testes width	‒	58‒81	67‒181
Testes-Ratio length: width	‒	1:1.7-1:2.1	1:3-1:5.4
Vaginal opening to anterior end	500‒830	462	526
Vaginal opening to genital pore	375‒630	362	447
Number of spines in MCO	16‒22	24	24
Number of spines in vestibule vaginal	‒	31	37
Reference	Yamaguti (1953)	Ramalingam (1960)	Ramalingam (1960)

Species	***P. minutum***	***P. carangis***
Hosts	*C. sexfasciatus*	*C. sansun*
Location	India	India
Total length	1500	2540‒3100
Total Width	300	460‒540
Haptor width	239	‒
Right side of haptoral lappet length	**65**	‒
Left side of haptoral lappet length	**65**	‒
Central haptoral lappet length	**119**	‒
Haptoral lappet width	360	320‒410
Clamps length	52	48
Number of testes	34	20‒24
Testes length	14	‒
Testes width	34‒44	‒
Testes-Ratio length: width	1:2.4-1:3.1	‒
Vaginal opening to anterior end	304	‒
Vaginal opening to genital pore	260	‒
Number of spines in MCO	12	22
Number of spines in vestibule vaginal	19	14‒16
Reference	Ramalingam (1960)	Ramalingam (1960)

* Data obtained from the average length of the four clamps.

Values in **bold** were taken of the line drawings.

MCO = male copulatory organ.

**Table 5:** Measurements (in µm) of haptoral armature (anchors, and hooks) of *Protomicrocotyle veracruzensis* sp. nov.

	Right lateral anchor (Ac1)	Left lateral anchor (Ac1’)	Right medial anchor (Ac2)	Left medial anchor (Ac2’)	Right central hook (Ho)	Left central hook (Ho’)
Total length (a)	43 (38‒50, n = 24)	42 (40‒47, n = 24)	27 (25‒29, n = 20)	27 (24‒29, n = 20)	18 (17‒19, n = 22)	18 (17‒19, n = 22)
Total width (b)	5 (5‒7, n = 24)	6 (5‒7, n = 24)	4 (2‒5, n = 22)	3 (2‒4, n = 20)	2 (2‒2, n = 22)	2 (2‒2, n = 22)
Opening length (c)	10 (7‒13, n = 24)	10 (7‒13, n = 23)	5 (4‒8, n = 22)	6 (4‒11, n = 20)	-	-
Internal root length (d)	16 (11‒19, n = 24)	15 (11‒17, n = 24)	7 (5‒11, n = 22)	9 (7‒12, n = 20)	-	-
Internal root width (e)	7 (5‒10, n = 24)	7 (5‒8, n = 24)	3 (2‒4, n = 22)	3 (2‒5, n = 20)	-	-
External root length (f)	16 (13‒20, n = 24)	15 (12‒19, n = 24)	9 (7‒13, n = 22)	9 (7‒11, n = 20)	-	-
External blade length (g)	23 (18‒29, n = 24)	24 (17‒34, n = 24)	16 (13‒19, n = 22)	18 (14‒20, n = 20)	-	-
Internal blade length (h)	20 (16‒25, n = 24)	20 (13‒31, n = 24)	13 (11‒17, n = 22)	15 (12‒20, n = 20)	-	-
Point length (i)	13 (5‒19, n = 24)	15 (8‒24, n = 24)	9 (5‒12, n = 22)	11 (8‒13, n = 20)	-	-

The letters in the parenthesis are represented in Figure 2.

*n*: number of specimens measured.

*Body* (Figures 2A, 5A, 7A). Body narrowly fusiform, 3887 (3209‒4709, n = 24) long (including haptoral lappet) and 804 (586‒1098, n = 24) wide at level of middle of testicular field (Figure 5A). Tegument with annular ridges posterior to the ovary that extend to posterior region of body, reaching the haptoral lappet (Figure 5A-a inset). Prohaptoral suckers anterolateral, muscular, oval; right oral sucker 76 (47‒97, n = 24) long, 48 (32‒59, n = 24) wide, and left sucker 82 (67‒94, n = 24) long, 49 (32‒58, n = 24) wide (Figures 2A, 5B). Buccal cavity subterminal, ventral, 35 (25‒44, n = 24) long, 52 (38‒72, n = 24) wide. Pharynx 54 (46‒58, n = 24) long, 52 (48‒59, n = 24) wide, muscular, and orbicular in shape. Esophagus long, with lateral diverticula. Cecal bifurcation posterior to male copulatory organ (MCO), 285 (190‒405, n = 24) from anterior end of body. Intestinal ceca lateral to midline and reproductive organs, extending into haptor; ceca with numerous short and lateral ramifications (Figures 2A, 5A).

*Haptor* (Figures 2A, 2C, 5A, 5E, 7A, 7C). Haptor asymmetrical, comprised of four lateral clamps on the left side and a terminal haptoral lappet; (no asymmetry of these structures observed); 630 (515‒685, n = 22) long, 575 (415‒980, n = 24) wide. Clamps located ventrally, each with a short peduncle (Figures 2A, 5A, 5C), positioned on left side of worm (on right side of drawing of worm in ventral view). First clamp (anterior to haptoral groove) 65 (46‒74, n = 22) long, 54 (32‒74, n = 22) wide; second clamp 65 (48‒76, n = 20) long, 58 (42‒76, n = 20) wide; third clamp 63 (49‒76, n = 22) long, 57 (42‒72, n = 22) wide; fourth clamp 63 (46‒72, n = 22) long, 57 (38‒76, n = 22) wide. Clamps typical of Gastrocotylidae; each clamp formed by a ventral arm of median spring, accessory skeletal piece, ventral and dorsal jaws of right side, ventral and dorsal jaws of left side, dorsal arm of dorsal jaw, dorsal arm of ventral jaw and ventral and dorsal oblique sclerite (Figure 5B). Small haptoral groove, with raised edges, between the first and second clamps (Figures 2A, 5A, 7A, 7C). Haptoral lappet elongate ovate, wider than long. Length of lappet measured in three regions (Figure 2A); right side of haptoral lappet (rt) 173 (136‒240, n = 22) long, middle region of haptoral lappet (mid) 206 (164‒259, n = 22) long, left side of haptoral lappet (lf) 194 (124‒259, n = 22) long. Haptoral lappet 769 (580‒920, n = 22) wide (Figure 2A), armed with two pairs of anchors, and one pair of hooks (Figures 2A, 2C, 5A, 5C, 5E, 7C; Table 5).

*Male reproductive structures* (Figures 5A, 6A, 6B, 7A, 7B). Testes 47 (36‒69, n = 23) in number, depressed elliptoid (and some are shallowly elliptoid), intercecal, anterior to descending ducts and germarium, arranged in two parallel fields, each field with one or two interspersed testes wide, forming an irregular column. Testes 47 (31‒59, n = 24) long, 142 (96‒176, n = 24) wide (Figure 5A), shallowly-doliform to shallowly-elliptoid in shape. Vas deferens dorsal to testes, running in zigzag pattern from anterior part of testes to MCO. Male copulatory organ 83 (74‒91, n = 24) long, 68 (55‒82, n = 24) wide, subspherical (Figures 3A, 6A, 6B, 7A, 7B), armed with 23 (16‒28, n = 23) spines. Spines of MCO hooklike, 43 (38‒47, n = 24) long, 3 (2‒5, n = 24) wide, arranged in a circle on anterior part of MCO (Figure 6A, 6B). Each spine with small knob in the anterior region just posterior to curved tip, knob and curved tip directed outwards (Figure 6B). Genital atrium ventral, near midline, anterior to cecal bifurcation, opening at 506 (340‒615, n = 24) from anterior end of body (Figure 5A).

*Female reproductive structures* (Figures 5A, 6C, 6E, 7A, 7B). Germarium intercecal, post-testicular, comprised of germarial bulb with immature oöcytes, 161 (133‒197, n = 24) long, 188 (149‒232, n = 24) wide, with a wide ascending duct and an irregularly descending duct that form loops that contain mature oöcytes. Descending duct united to the oötype just anterior to the ovary (Figure 6E). Vaginal duct/seminal receptacle and vitelline duct connect to oötype; uterus ascends from oötype to the genital atrium (Figure 6E). Vaginal pore ventral, anterior to vaginal vestibule. Vaginal vestibule 92 (78‒103, n = 24) long, 72 (49‒96, n = 24) wide (Figures 5A, 6C, 6D, 7A, 7B), located 610 (440‒730, n = 24) from anterior end of body and 203 (130‒225, n = 24) from the genital pore, lateral to midline on the side opposite to that having the haptoral clamps. Vaginal vestibule armed with 49 (33‒59, n = 23) spines 42 (38‒46, n = 24) long, 4 (2‒5, n = 24) wide; basal spines smallest, uppermost spines largest (Figure 6C). Each spine elongated in shape with 5 (4‒6, n = 24) small needle-like spikes at the distal end (Figure 6D). Vaginal duct descends from vaginal vestibule to germarium; small elongate seminal receptacle 25 (18‒33, n = 4) long, 15 (8‒21, n = 4) wide, at terminal end of vaginal duct, connected to ventral side of germarium (Figure 6E). Vitelline glands in two lateral fields, starting just posterior to vaginal vestibule, overlapping ceca anteriorly and posteriorly and the lateral margin of the testes, uniting posterior to germarium, extending to posterior region of body but not reaching the haptoral lappet (Figures 2A, 5A, 7A, 7C). Vitellogenic ducts from each field unite in the region dorsal to the germarium, forming a single vitelline channel that leads to and connects to the oötype (Figures 5A, 6E).

*Eggs* (Figure 6F). Uterus contains 3 (1‒8, n = 13) eggs. Eggs elongate, elliptoid, with long polar filaments at each pole; length, not including polar filaments, 182 (128‒229, n = 15), 57 (47‒72, n = 15) wide. Filament in anterior end (as positioned in uterus) 237 (210‒250, n = 7) long, 5 (4‒6, n = 7) wide, and posterior end 191 (137‒247, n = 5) long, 4 (2‒6, n = 5) wide.

### Taxonomic summary

**Type host:***Caranx latus* (Carangidae).

**Common name:** Horse-eye jack.

**Type locality:** Littoral waters of Gulf of Mexico off Casitas (20° 15' 31.5” N; 96° 47'49.5” W), Veracruz, Mexico; collected in May 2005.

**Site of infection:** Gill filaments

**Other locality:** Littoral waters of Gulf of Mexico off Puerto de Veracruz (19° 13' 11.2” N; 96° 09' 24.4” W), Veracruz, Mexico; collected in May 2022.

**Etymology:** The specific epithet is derived from the name of the state of Veracruz, Mexico, where these specimens were collected.

**Abundance, prevalence, mean intensity of infection and range of intensity:** General infection characterization: *C. latus*, 12.17 of abundance, 2 infected fish of 6 examined (33.33%), 36.50 and 14‒59. Casitas: 2.80 of abundance, 1 infected fish of 5 examined (20%), 14 and 14; Puerto de Veracruz: 59 of abundance, 1 infected fish of 1 examined (100%), 59 and 59.

**Specimen deposit:** holotype CNHE-12819; paratypes CNHE-12820 to 12821, HWML-216978 to 216984 and 2166998, CHE-P00148.

**ZooBank registration:** D3E641BF-5A28-447E-A097-47813D3CA67C

**Genbank accession numbers:** Cytochrome C Oxidase Subunit I (CO1) OR282833 to OR282836; 28S rDNA OR282895 to OR282898.

### Remarks

Within the suite of characters of the genus that were mentioned above, several differences in character states have been used to distinguish between species of *Protomicrocotyle*: the number of testes (Yamaguti, 1953; Bravo-Hollis, 1966; Pillai & Pillai, 1978); the number and shape of spines on the male copulatory organ (Yamaguti, 1953; Ramalingam, 1960; Pillai & Pillai, 1978; Kritsky et al., 2011); and the number of vaginal spines (Ramalingam, 1960; Wahl, 1972; Pillai & Pillai, 1978). Employing these characters, *P. veracruzensis* sp. nov. has 47 (36‒69 testes; *P. mirabilis* 27 (23–33 testes) (Kritsky et al., 2011), *P. carangis* (20–24) (Pillai & Pillai, 1978), *P. manteri* (22–33) (Bravo-Hollis, 1989), *P. minutum* (34) (Ramalingam, 1960) have less testes and the new species and *P. celebesensis* (40–57) (Yamaguti, 1953), *P. ivoriensis* (50) (Wahl, 1972), *P. madrasensis* (64) (Ramalingam, 1960), *P. mannarensis* (42–52) (Ramalingam, 1960) and *P. nayaritensis* (46–48) (Bravo-Hollis, 1979) appear to have a number of testes that overlap with that of the new species, although detailed data is lacking in some species of each group. The male copulatory organ of *P. veracruzensis* sp. nov. has 23 (16–28) spines: the MCO of *P. minutum* has less (12 spines) (Ramalingam, 1960) and *P. manteri* and *P. nayaritensis* have more (33–38 and 48–54 spines, respectively); for the other species, *P. carangis* (22), *P. celebesensis* (16–22), *P. ivoriensis* (25–26), *P. madrasensis* (24), *P. mannarensis* (24), and *P. mirabilis* (16–21), the number of spines overlaps with that of the new species. The vagina of the new species has 49 (33‒59) spines; *P. carangis* (14–16), *P. madrasensis* (31), *P. mannarensis* (37), *P. manteri* (15), and *P. minutum* (19) all have less vaginal spines, *P. mirabilis* (49) and *P. nayaritensis* (22–46) have numbers of vaginal spines that overlap with the new species, and there is no information for *P. celebesensis* and *P. ivoriensis*.

Some authors have mentioned the arrangement of the testes in their descriptions, but often they have mention only that the testes are arranged in two fields/rows (Yamaguti, 1953; Ramalingam, 1960; Pillai & Pillai, 1978; Bravo-Hollis, 1979). The testes of *P. mirabilis*, *P. carangis*, and *P. mannarensis* are arranged in a single row on each side of the midline (MacCallum, 1918; Ramalingam, 1960; Wahl, 1972; Pillai & Pillai, 1978; Kritsky et al., 2011). The testes of *P. veracruzensis* sp. nov., *P. minutum*, and *P. nayaritensis* are arranged in rows with 1–2 adjacent testes on each side (Ramalingam, 1960; Bravo-Hollis, 1966), in *P. madrasensis* they appear to be in row with 2 adjacent testes on each side (Ramalingam, 1960), and those of *P. ivoriensis* have rows with 1–3 adjacent testes (Wahl, 1972).

Of the known species of *Protomicrocotyle,* the new species is most similar to the species from the Atlantic Ocean basin, *P. mirabilis* and *P. ivoriensis*. Using the structures mentioned above, the new species can be distinguished from *P. mirabilis* by having more testes (36‒69 vs. 23–33, respectively); there is overlap in the number of testes of *P. ivoriensis* (36‒69 vs. 50, respectively) by having less testes, although, if the number of testes reported for the latter species was invariant, or an average, is not known. The new species also can be distinguished from these two species by the arrangement of the testes; the new species has 1–2 adjacent testes in the lateral rows vs. *P. mirabilis*, which has testes in a single-file row in each field and *P. ivoriensis* has groups of 1–3 adjacent testes in each row. The relation between the length and width (L: W) of the testes in the new species is L: W: 1:3 and in *P. mirabilis* is L: W: 1:1.1, showing that the testes of the new species are larger than those of *P*. *mirabilis*. The relationship between the length and width of the testes of the new species is also greater than in *P. ivoriensis* (1:3 vs. 1:1.6), *P. madrasensis* (1:3 vs. 1:1.7-2.1), *P. manteri* (1:3 vs. 1:1.8), *P*. *nayaritensis* (1:3 vs. 1:1.4), smaller than *P. mannarensis* (1:3 vs. 1:3-5.41) and in range with *P. minutum* (1:3 vs. 1:2.4-3.1). Complete detailed comparative data for the new species and the other nine valid species is given in Table 4.

### Morphometric analyses

Results of the principal components analysis (PCA) show an accumulated variance of 52.75 (Table 6A) of the first two components and the morphological variables that most influence the multidimensional arrangement of the first component. They are, in order of importance: the width of the body, the width of the ovary, the width of the haptor, the average width of the testes, the distance from the vaginal vestibule to the genital pore and the total longitude of the body. The variables that most influenced the second component are, in order of importance: the number of spines of the vaginal vestibule and the length of the spines of the vaginal vestibule. Eigenvalues and accumulated variance for first two principal components are presented in Table 6A.

**Table 6:** Cumulative variance of (**A**) the first two principal components and (**B**) factor values of discriminant analyses.

**A**			
Principal Component	Eigenvalue	% variance	% Accumulate variance
1	0.81	44.10	44.10
2	0.16	8.651	52.75
			

**B**			
Factor	Eigenvalue	% variance	% Accumulate variance
1	55.73	46.88	46.88
2	49.49	41.64	88.52

**Figure 8:**
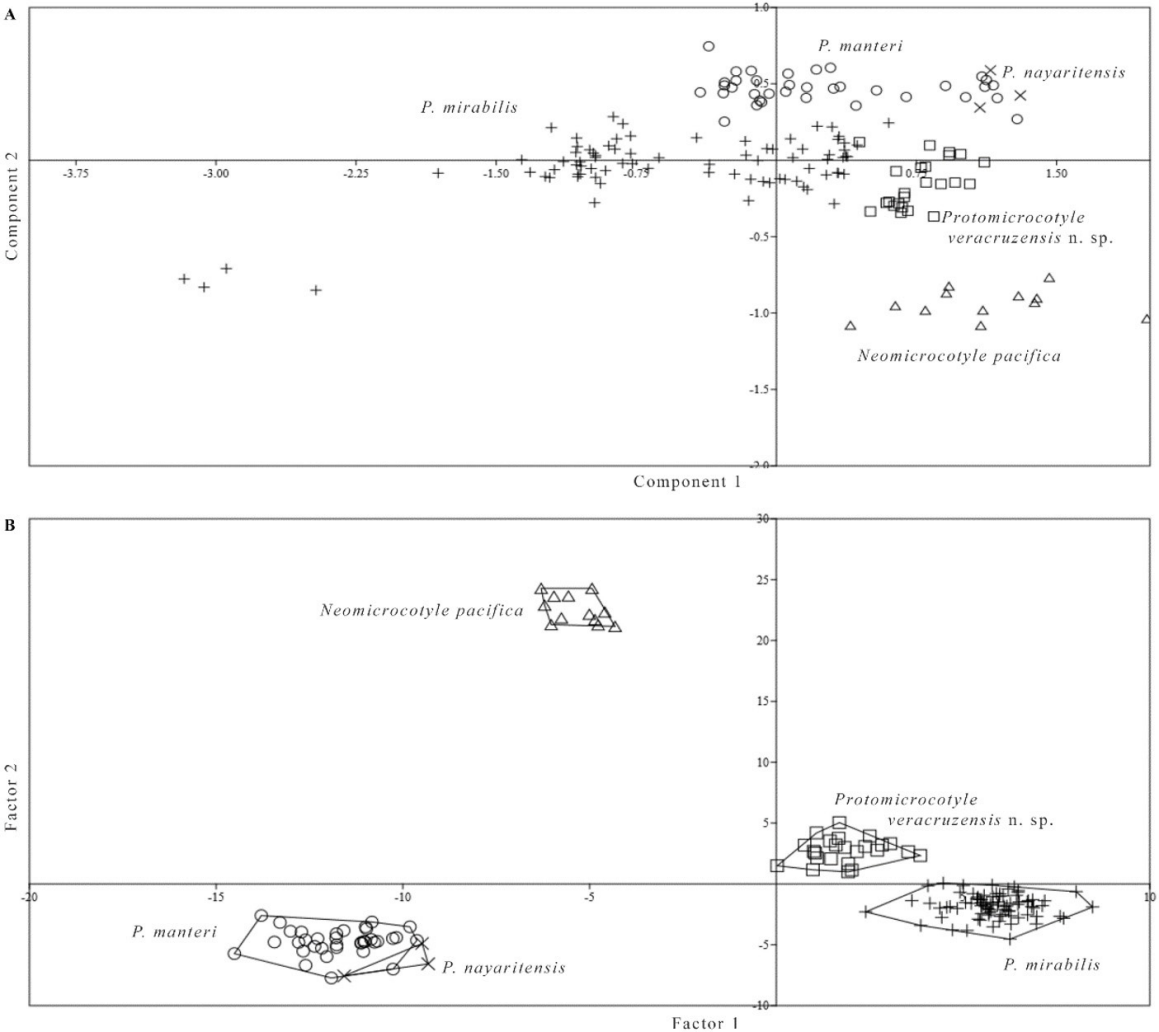
Morphometric analyses of the characteristics of species of *Protomicrocotyle*. A. Principal components analyses. B. Discriminant analyses.

In the Discriminant Analysis (DA), the first two factor axes explained 88.52% of the total variation (Table 6B). The first factor (eigenvalue 55.73, 46.88 of variation) alone was the main discriminant function, which arranged the specimens of *Protomicrocotyle* and *Neomicrocotyle* with the morphological variables, in order of importance: the number of spines in the male copulatory organ and the length of the spines of the vaginal vestibule. The second factor (eigenvalue 49.49, 41.64 of variation) has a value almost equal to the first factor, and separated the specimens of *Protomicrocotyle* and *Neomicrocotyle* with the morphological variables, in order of importance: the width of the body, number of spines of the vaginal vestibule, number of testes, longitude of the male copulatory organ and the width of the ovary. The DA, like the PCA, in the multidimensional plane, separated the five groups that correspond to the specimens of *P. mirabilis*, *P. manteri*, *P. nayaritensis*, *Neomicrocotyle pacifica* and the new species, *P. veracruzensis* sp. nov. (Figure 8A, 8B). An important finding was that Factor 1, of the DA, separated the specimens of the Atlantic species (*P. mirabilis* and *P. veracruzensis* sp. nov.) from the specimens of the Pacific species (*P. manteri*, *P. nayaritensis*, and *Neomicrocotyle pacifica*). Finally, Factor 2, separated the specimens of *Protomicrocotyle* from *Neomicrocotyle pacifica* (Figure 8B).

### Molecular study

Sequences (16 of CO1 and 14 of 28S), belonging to *Protomicrocotyle mirabilis* (OR282821–OR282832; OR282885–OR282894) and the new species (OR282833–OR282836; OR282895–OR282898) were obtained in the present study (Table 2). The sequences of CO1 and sequences of 28S from GenBank® (Sayers et al., 2020) of *Bilaterocotyloides carangis* Ramalingam, 1961, *Bilaterocotyloides madrasensis* Radha, 1966, and *Allodiscocotyla diacanthi* Unnithan, 1962 were used in the distance’s genetic analyses and Neighbor-Joining (Table 2). The final alignment of CO1 consisted in 20 sequences, with a length of 403 bp. The 14 sequences of 28S were aligned with 8 sequences of 28S from GenBank (*Neomicrocotyle pacifica* (Meserve, 1938) Yamaguti, 1968; *Neomicrocotyle* sp.; *Lethacotyle vera*Justine, Rahmouni, Gey, Schoelinck & Hoberg, 2013; *B. carangis*; *B. madrasensis*; and *A. diacanthi*) (Table 2). The final alignment consisted of the 22 partial sequences of the 28S region, with a length of 750 bp.

**Figure 9:**
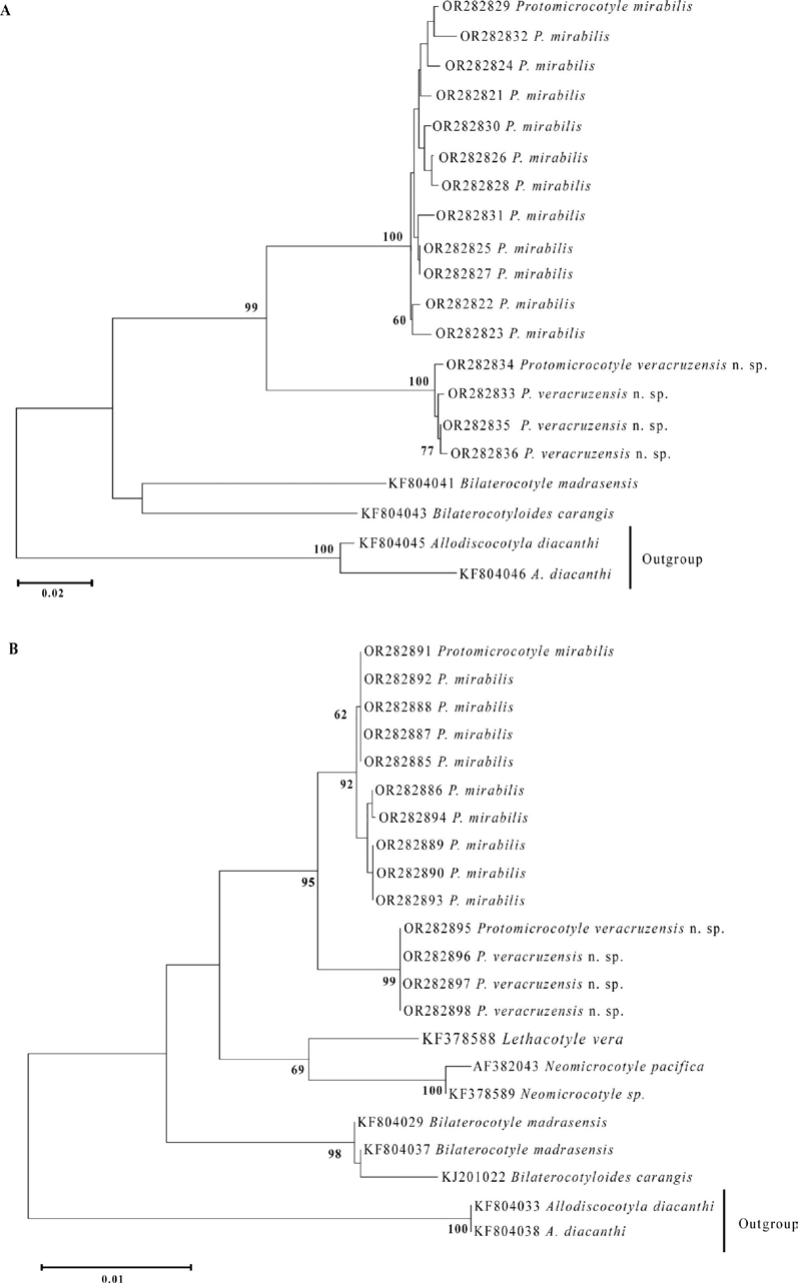
Cluster analysis showing similarities between specimens of *Protomicrocotyle* and other protomicrocotylids. A. Dendrogram of sequences of cytochrome c oxidase subunit I. B. Dendrogram of sequences of 28S rDNA. Note: Values in bold are the bootstrap values.

The intraspecific genetic variation between specimens of *P. mirabilis* was 0% to 2.01% for CO1 and 0% to 0.14% for 28S. Between specimens of *P. veracruzensis* sp. nov., the variation was 0.25% to 0.75% for CO1 and 0% for 28S. The interspecific genetic variation between specimens of *P. mirabilis* and *P. veracruzensis* sp. nov. was 8.48% to 10.53% with CO1, and 0.81% to 0.95% with 28S (Figure 9A, 9B; Table 77B).

**Table 7:** Intraspecific and interspecific genetic distances estimated for (**A**) mitochondrial cytochrome c oxidase subunit I (CO1) and (**B**) 28S rDNA of *Protomicrocotyle veracruzensis* sp. nov. and other species of Protomicrocotylidae. Pairwise corrected *p-distances* are expressed as percentages (%).

**A**								
	KF804045	KF804046	KF804041	KF804043	OR282821*	OR282822*	OR282823*	OR282824*
KF804045								
KF804046	3.55%							
KF804041	19.86%	22.34%						
KF804043	18.09%	20.21%	12.41%					
OR282821*	20.21%	23.05%	16.67%	15.25%				
OR282822*	20.21%	23.05%	16.31%	14.89%	**1.00%**			
OR282823*	20.21%	23.05%	16.31%	14.89%	**1.24%**	**0.74%**		
OR282824*	20.92%	23.76%	16.31%	14.89%	**0.77%**	**1.28%**	**1.28%**	
OR282825*	20.21%	23.05%	16.31%	14.89%	**0.50%**	**0.50%**	**0.74%**	**0.77%**
OR282826*	20.21%	23.05%	16.31%	15.25%	**0.75%**	**0.75%**	**1.00%**	**1.02%**
OR282827*	20.21%	23.05%	16.31%	14.89%	**0.50%**	**0.50%**	**0.75%**	**0.77%**
OR282828*	20.21%	23.05%	16.31%	15.25%	**1.00%**	**0.99%**	**1.24%**	**1.28%**
OR282829*	20.57%	23.40%	16.67%	15.25%	**1.00%**	**0.99%**	**1.24%**	**0.77%**
OR282830*	20.21%	23.05%	16.31%	14.89%	**0.75%**	**0.74%**	**0.99%**	**1.02%**
OR282831*	19.86%	22.70%	16.67%	14.54%	**1.00%**	**1.00%**	**1.25%**	**1.29%**
OR282832*	20.92%	23.76%	16.31%	15.60%	**1.26%**	**1.75%**	**2.01%**	**1.03%**
OR282833**	20.57%	23.40%	15.60%	16.31%	*9.20%*	*8.71%*	*9.45%*	*9.21%*
OR282834**	20.92%	23.76%	15.25%	16.67%	*9.45%*	*8.68%*	*9.43%*	*9.46%*
OR282835**	20.92%	23.76%	15.25%	16.67%	*8.96%*	*8.68%*	*9.43%*	*8.95%*
OR282836**	20.92%	23.76%	15.25%	16.67%	*9.20%*	*8.93%*	*9.68%*	*9.21%*

	OR282825*	OR282826*	OR282827*	OR282828*	OR282829*	OR282830*	OR282831*	OR282832*
OR282826*	**0.75%**							
OR282827*	**0.00%**	**0.75%**						
OR282828*	**0.99%**	**0.25%**	**1.00%**					
OR282829*	**0.99%**	**0.75%**	**1.00%**	**0.74%**				
OR282830*	**0.74%**	**0.50%**	**0.75%**	**0.50%**	**0.50%**			
OR282831*	**0.50%**	**1.26%**	**0.50%**	**1.00%**	**1.25%**	**1.00%**		
OR282832*	**1.25%**	**1.52%**	**1.26%**	**1.50%**	**0.75%**	**1.25%**	**1.50%**	
OR282833**	*8.71%*	*9.52%*	*8.73%*	*9.70%*	*9.70%*	*9.45%*	*9.27%*	*10.05%*
OR282834**	*9.18%*	*9.27%*	*8.98%*	*9.68%*	*9.68%*	*9.43%*	*9.75%*	*10.53%*
OR282835**	*8.68%*	*9.27%*	*8.48%*	*9.68%*	*9.68%*	*9.43%*	*9.25%*	*10.03%*
OR282836**	*8.93%*	*9.52%*	*8.73%*	*9.93%*	*9.93%*	*9.68%*	*9.50%*	*10.28%*

	OR282833**	OR282834**		OR282835**		OR282836**	
OR282834**	**0.75%**					
OR282835**	**0.75%**	**0.50%**				
OR282836**	**0.75%**	**0.74%**	**0.25%**			

**B**								
	KF804033	KF804038	KF804029	KF804037	KJ201022	AF382043	KF378589	KF378588
KF804033								
KF804038	0.00%							
KF804029	5.25%	5.25%						
KF804037	5.25%	5.25%	0.00%					
KJ201022	5.29%	5.29%	0.44%	0.44%				
AF382043	6.06%	6.06%	2.42%	2.42%	2.94%			
KF378589	5.93%	5.93%	2.20%	2.20%	2.79%	0.14%		
KF378588	6.21%	6.21%	3.03%	3.03%	3.38%	1.79%	1.65%	
OR282885*	5.25%	5.25%	2.55%	2.55%	3.23%	2.69%	2.48%	2.20%
OR282886*	5.14%	5.14%	2.43%	2.43%	3.10%	2.83%	2.63%	2.35%
OR282887*	5.26%	5.26%	2.56%	2.56%	3.24%	2.69%	2.48%	2.21%
OR282888*	5.26%	5.26%	2.56%	2.56%	3.24%	2.70%	2.48%	2.21%
OR282889*	5.15%	5.15%	2.44%	2.44%	3.08%	2.84%	2.62%	2.34%
OR282890*	5.17%	5.17%	2.45%	2.45%	3.09%	2.85%	2.62%	2.34%
OR282891*	5.31%	5.31%	2.59%	2.59%	3.23%	2.72%	2.48%	2.20%
OR282892*	5.27%	5.27%	2.56%	2.56%	3.23%	2.70%	2.48%	2.20%
OR282893*	5.20%	5.20%	2.46%	2.46%	3.10%	2.87%	2.63%	2.35%
OR282894*	5.16%	5.16%	2.44%	2.44%	3.09%	2.85%	2.62%	2.34%
OR282895**	5.38%	5.38%	2.96%	2.96%	3.67%	2.82%	2.62%	2.48%
OR282896**	5.38%	5.38%	2.96%	2.96%	3.67%	2.82%	2.62%	2.48%
OR282897**	5.38%	5.38%	2.96%	2.96%	3.67%	2.82%	2.62%	2.48%
OR282898**	5.41%	5.41%	2.97%	2.97%	3.68%	2.83%	2.62%	2.48%

	OR282885*		OR282886*		OR282887*		OR282888*		OR282889*		OR282890*		OR282891*		OR282892*	
OR282886*		**0.13%**															
OR282887*		**0.00%**		**0.13%**													
OR282888*		**0.00%**		**0.14%**		**0.00%**											
OR282889*		**0.14%**		**0.00%**		**0.14%**		**0.14%**									
OR282890*		**0.14%**		**0.00%**		**0.14%**		**0.14%**		**0.00%**							
OR282891*		**0.00%**		**0.14%**		**0.00%**		**0.00%**		**0.14%**		**0.14%**					
OR282892*		**0.00%**		**0.14%**		**0.00%**		**0.00%**		**0.14%**		**0.14%**		**0.00%**			
OR282893*		**0.14%**		**0.00%**		**0.14%**		**0.14%**		**0.00%**		**0.00%**		**0.14%**		**0.14%**	
OR282894*		**0.14%**		**0.00%**		**0.14%**		**0.14%**		**0.14%**		**0.14%**		**0.14%**		**0.14%**	
OR282895**		*0.81%*		*0.94%*		*0.81%*		*0.81%*		*0.95%*		*0.95%*		*0.82%*		*0.81%*	
OR282896**		*0.81%*		*0.94%*		*0.81%*		*0.81%*		*0.95%*		*0.95%*		*0.82%*		*0.81%*	
OR282897**		*0.81%*		*0.94%*		*0.81%*		*0.81%*		*0.95%*		*0.95%*		*0.82%*		*0.81%*	
OR282898**		*0.81%*		*0.95%*		*0.81%*		*0.81%*		*0.95%*		*0.95%*		*0.82%*		*0.81%*	

	OR282893*	OR282894*	OR282895**	OR282896**	OR282897**	OR282898**
OR282894*	**0.14%**					
OR282895**	*0.96%*	*0.95%*				
OR282896**	*0.96%*	*0.95%*	**0.00%**			
OR282897**	*0.96%*	*0.95%*	**0.00%**	**0.00%**		
OR282898**	*0.96%*	*0.95%*	**0.00%**	**0.00%**	**0.00%**	

The values in **bold** represents the intraspecific divergence in *Protomicrocotyle mirabilis* and *P. veracruzensis* sp. nov;

The values in *italics* represents the interspecific divergence between *P*. *mirabilis* and *P*. *veracruzensis* sp. nov.

* Sequences of *Protomicrocotyle mirabilis*;

** Sequences of *Protomicrocotyle veracruzensis* sp. nov.

The NJ analysis using CO1 and 28S sequences of all species of Protomicrocotylidae currently in GenBank and *Allodiscocotyla diacanthi* from GenBank as outgroup (Table 2) resulted in the identification of a single group of *Protomicrocotyle* with support of 99% with CO1 and 95% with 28S (Figure 9A, 9B). *Protomicrocotyle mirabilis* and *P*. *veracruzensis* sp. nov. form each one an independent group with the support of 100% with CO1 (Figure 9A), and 92% and 99% with 28S (Figure 9B), respectively. *Protomicrocotyle* nested within members of the family Protomicrocotylidae with high support with both molecular markers (Figure 9A, 9B).

## Discussion

As with many groups of helminths, the time span since the first member of the group was described by MacCallum (1918) and today’s descriptions encompass many advances in the development of our understanding of the characteristics that reveal the limits between species. For groups that are not well-studied, like the genus *Protomicrocotyle*, a suite of characters are most useful for the identification of members on a group level instead of a single diagnostic feature. This has resulted in descriptions which mention only the characters that are known at that time in order to distinguish the new species from a particular congener. The same features, as the number of testes, the number of spines in the MCO, and the number of spines in the vagina were used to distinguish the new species from others of the genus. However, these and other values often overlapped with particular species in such a way that no single character could distinguish the new species from the other species of *Protomicrocotyle*. For better understanding of the intra- and interspecific variation, Table 4, with the measurements of the 10 known species, was developed and included herein. Most of the measurements of the previously known species were taken from published descriptions (see Table 4 for citations of the works that were consulted). Possibly because some authors were only reporting a new locality or host record, many of those articles did not provide a full description, only mentioning those characters that helped them assign their material. The limitations of that material and those descriptions were not sufficient to confirm the assignment of those specimens to a particular species (Table 3). In those cases, new material and molecular studies will be needed for confirmation of their identity.

One area of interest is in the use of the measurements of the hard parts (hooks, anchors, spines, etc.) as informative characters in descriptions of new taxa; a few examples of the use of the measurements of these structures in recent studies of monogeneans are mentioned herein. Vaughan & Christison (2012), proposed a measurement scheme for the haptoral armament of members of *Callorhynchocotyle* Suriano & Incorvaia, 1982 and used the measurements in a systematic study of the members of that genus. Most recently, Vaughan et al. (2021) used the same scheme and provided the measurements of the haptoral armament of *Scyliorhinocotyle narvaezae* Vaughn, Christison & Hansen, 2021. These works prompted us to provide the measurements for the haptoral armament in this study (Table 5). It is hoped that similar measurement data will be provided by authors in future descriptions of new species of *Protomicrocotyle*.

In the present study, the seminal receptacle of *P. veracruzensis* sp. nov. was observed and described, although it could not be distinguished in all specimens. This structure often is not mentioned in descriptions of other members of the genus. In most cases, whether the structure is present or not, or whether it was just not mention, cannot be determined. However, Ramalingam (1960) described and illustrated the seminal receptacles of *P. madrasensis*, *P. mannarensis*, and *P. minutum*. A review detailed of the material of the remaining members of the genus is necessary in order to verify the presence or absence of the seminal receptacle in those species. In most cases, collections of new material will be required.

In a similar manner, many authors have not mentioned whether the clamps are sessile or pedunculate. Because the data is incomplete or conflicting, this feature was not used in the comparisons. Of the nine previously-known species, four authors have reported that three species of *Protomicrocotyle* have clamps with peduncles: *P. nayaritensis*, *P. mirabilis*, and *P. manteri* [see Bravo-Hollis (1979), Caballero y Caballero & Bravo-Hollis (1965), MacCallum (1918), and Bravo-Hollis (1966), respectively]. Three authors have reported species that have clamps that are sessile: *P. mirabilis*, by Kritsky et al. (2011), contrary to previous reports by Caballero y Caballero & Bravo-Hollis (1965) and MacCallum (1918); *P. madrasensis*, *P. mannarensis*, and *P. minutum* by Ramalingam (1960); and *P. carangis* by Pillai & Pillai (1978). The presence or absence of peduncles for *P. mirabilis* was not mentioned by Johnston & Tiegs (1922), Koratha (1955), Wahl (1972), and Bravo-Hollis (1989), and for *P. celebesensis* by Yamaguti (1953), and Barton et al. (2009). Wahl (1972) did not mention peduncles for *P. ivoriensis*; however, in the drawing of *P. ivoriensis* the clamps are shown to have peduncles [Wahl (1972), his Figure 2a]. The clamps of the new species, *P. veracruzensis* sp. nov., have peduncles similar to those of *P. ivoriensis*, and specimens of *P. mirabilis* reported as part of this study had clamps with peduncles. In all these species, the peduncles are short [as mentioned by MacCallum (1918)], which might explain why they were not given emphasis by some authors.

Finally, the orientation of the clamps has been mentioned only by Kritsky et al. (2011), who said that they could be dextral or sinistral, but they did not mention their relationship to other structures. The clamps of specimens of *P. mirabilis* and *P. veracruzensis* sp. nov., were observed to be on the right in some worms and on the left side of other worms, but always they were opposite to the vaginal vestibule. The male copulatory organ also always was opposite to the vaginal vestibule, and always on the same side as the clamps. This has not been mentioned by previous author.

In Mexico, two species of *Protomicrocotyle* have been described, *P. manteri* and *P. nayaritensis* in the Pacific Ocean (Bravo-Hollis, 1966, 1979), and one, *P. mirabilis*, has been recorded in the Gulf of Mexico (Caballero y Caballero & Bravo-Hollis, 1965; Kritsky et al., 2011); other reports from the Caribbean Sea (Caballero y Caballero & Bravo-Hollis, 1967; Bravo-Hollis, 1989; Kritsky et al., 2011) and from Ivory Coast, Africa (Wahl, 1972) could not be evaluated properly because new material must be collected for comparative morphological and molecular studies.

This work adds a fourth species reported from Mexico; in this study, *P. mirabilis* and *P. veracruzensis* sp. nov. are the first members of the genus to be characterized with morphological and molecular data. With this new species, tenth member to the genus, the helminthological record of monogenean parasites of *C. latus* is now seven species: *Ahpua piscicola* Caballero y Caballero & Bravo-Hollis, 1973, *Allopyragraphorus winteri* (Caballero y Caballero & Bravo-Hollis, 1965) Bravo-Hollis & Salgado-Maldonado, 1983, *Cemocotyle noveboracensis* Price, 1962, *Cemocotylella elongata* (Meserve, 1938) Price, 1962, *P. mirabilis*, *P. veracruzensis* sp. nov., and *Pseudomazocraes selene* Hargis, 1957. Fifteen species have been reported from *C. hippos: Axine* sp., *Ahpua piscicola*, *Allopyragraphorus caballeroi* (Zerecero, 1960) Yamaguti, 1963, *A. hippos* (Hargis, 1956) Yamaguti, 1963, *A. winteri*, *Cemocotyle carangis* (MacCallum, 1913) Sproston, 1946, *C. noveboracensis* Price, 1962, *Cemocotylella elongata* (Meserve, 1938) Price, 1962, *N. pacifica*, *Pseudomazocraes monsivaisae* Caballero y Caballero & Bravo Hollis, 1955, *P. riojai* (Caballero y Caballero & Bravo-Hollis, 1963) Lebedev, 1970, *P. selene*, *P. mirabilis*, *Salinacotyle mexicana* (Caballero y Caballero & Bravo-Hollis, 1963) Lebedev, 1984 and, *Zeuxapta seriolae* (Meserve, 1938) Price, 1962 [see Mendoza-Garfias et al. (2017)].

The use of morphological and molecular information has been a useful tool in the description of new species of Monogenea (Aguiar et al., 2017; Camargo & Santos, 2020; Torres-Carrera et al., 2020; Zago et al., 2021; Ayadi et al., 2022; Dmitrieva et al., 2022), and the relevance of the integrative taxonomy approach in the description and redescription of species of helminth has recently been highlighted. This study, with the molecular characterization of the CO1 and 28S genes of *P. mirabilis* and *P. veracruzensis* sp. nov. can be added to this list.

The molecular analysis revealed that the level of interspecific genetic variation between *P. mirabilis* and *P. veracruzensis* sp. nov. with CO1 sequence data is distinctly higher than that of the interspecific genetic variation with 28S (Table 7), as one would expect; other studies show the same pattern of high interspecific genetic variation between members of species of the same genus (Ayadi et al., 2017; Camargo & Santos, 2020; Torres-Carrera et al., 2020). In the genus, *Microcotyle* Van Beneden & Hesse, 1863, intraspecific nucleotide divergence of 0% to 1.4% has been reported in *Microcotyle visa* Bouguerche, Gey, Justine & Tazerouti, 2019, and an interspecific difference of 9.5% to 10.7% between *Microcotyle isyebi* Bouguerche, Gey, Justine & Tazerouti, 2019 and *M*. *visa* (Bouguerche et al., 2019). However, percentages of genetic divergence within and among taxa cannot be interpreted until a more complete molecular database is available (Torres-Carrera et al., 2020). Even taking this into account, the use of morphological data and the combination of different molecular markers are convincing evidence sufficient to differentiate *P*. *veracruzensis* sp. nov. from the other known members of the genus. As well, the same integrative approach corroborates evidence of the presence of *P. mirabilis* in the localities sampled in this study and the previous report of the species from Tuxpan by (Caballero y Caballero & Bravo-Hollis, 1965) and Campeche (Caballero y Caballero & Bravo-Hollis, 1967).
